# Pathogenic autoantibodies targeting DNA repair proteins are shared by cancer patients and healthy children later diagnosed with cancer

**DOI:** 10.3389/fimmu.2026.1911932

**Published:** 2026-10-14

**Authors:** Anastassia Serguienko, Jeetesh Sharma, Ronas Shakya, Monica Cheng Munthe-Kaas, Kjetil Boye, Ola Myklebost

**Affiliations:** 1 Department of Clinical Science, University of Bergen, Bergen, Norway; 2 Department of Global Public Health and Primary Care, University of Bergen, Bergen, Norway; 3 The Norwegian Childhood Cancer Biobank, The Department of Pediatric Oncology and Hematology, Oslo University Hospital, Oslo, Norway; 4 Department of Oncology, Oslo University Hospital, Oslo, Norway; 5 Department of Tumor Biology, Institute for Cancer Research, Oslo University Hospital, Oslo, Norway; 6 Western Norway Familial Cancer Center, Haukeland University Hospital, Bergen, Norway

**Keywords:** ALSPAC, leukemia, neuroblastoma, sarcoma, TIGER domain, osteosarcoma, rhabdomyosarcoma

## Abstract

The etiology of pediatric cancer remains poorly understood and does not fit the classical view as an age-associated genetic disease, suggesting yet unknown mechanisms might be involved. Using high-density native proteome arrays, we analyzed sera from 375 healthy individuals and patients. We found a significant enrichment of autoantibodies against DNA repair proteins shared by cancer patients and healthy children diagnosed with cancer years later. These autoantibodies were detected in multiple cancer types and exhibited remarkable age-specificity. To explore their potential pathogenic significance, we assessed the ability of serum IgG to penetrate live cells. We confirmed that IgG can indeed enter live intact cells and identified a specific pattern of subcellular localization prevalent in pediatric patients. These findings offer a novel direction for the investigation of etiology of pediatric cancer and cancer in general.

## Introduction

Autoantibodies - antibodies recognizing the body’s own proteins - are common in healthy people but are also involved in autoimmune disorders (1, 2). They can be present at birth or can appear shortly after and increase toward older ages (3–6). Antinuclear autoantibodies at low concentrations have been reported in up to 40% of healthy people (7, 8). Although the repertoires are highly heterogeneous, many autoantibodies are shared by a large fraction of healthy individuals (1).

The pathogenic effects of autoantibodies are not limited to classical autoimmune diseases. In cancer patients, mutated proteins, so-called neoantigens, can trigger the generation of autoantibodies that cross-react with wild-type (wt) proteins, and wt proteins aberrantly expressed or post-translationally modified in the tumor can also induce autoantibodies (9). Such tumor-associated autoimmunity may give rise to paraneoplastic syndromes in distant tissues (10, 11). Both positive and negative associations between autoimmune diseases and cancer have been reported, including an interesting population-based study showing a positive association between all tested autoimmune conditions and gastric cancer (12). While the mechanistic aspects of tumor-induced autoimmunity are quite well characterized, the higher cancer incidence among patients with some autoimmune diseases has not been well explained, with a major role being attributed to inflammation (12, 13).

Pediatric cancers arise at ages when their tissues of origin undergo critical developmental processes and waves of proliferation (14). Despite the increased risk of mutations due to the large number of cell divisions in growing tissues, pediatric cancers are rare and characterized by a paucity of somatic mutations (15–17). Although genetic predisposition is thought to play an important role in early cancers, only 6-10% of patients have so far been shown to have likely pathogenic gene variants (18). Interestingly, novel mutation signatures caused by unknown mechanisms have recently been identified (15). Therefore, the understanding of the etiology of pediatric cancer remains unsatisfactory.

Using high-density human proteome arrays, we performed autoantibody profiling of serum samples collected at diagnosis from 175 patients with nine different cancer types and 141 healthy controls of corresponding ages. We found that the sera of pediatric patients with different cancer types were enriched in autoantibodies against DNA repair proteins. Strikingly, we found some of the most enriched autoantibodies also in the serum of healthy children who got cancer years later.

Our data suggest that these autoantibodies might be involved in cancer etiology, which is further supported by our finding of specific intracellular localization of patients’ serum IgG. Therefore, pathogenic penetrating autoantibodies may represent a novel mechanism of cancer predisposition.

## Materials and methods

### Patient samples

All patient and control groups are listed in **Supplementary Table S1**. The main cohort, which included 91 adult sarcoma and ALL patients and 84 pediatric patients, was obtained from the NoSarC Norwegian Sarcoma Study, a collection of tumor and blood samples from >700 sarcoma patients from all Norwegian health regions collected from 2014-2017, from the Leukemia Center at UiB, and from the Norwegian Childhood Cancer Biobank, Oslo. Additional pediatric sarcoma samples were provided by the Oslo University Hospital Cancer Research Biobank.

Anonymous control samples were obtained from blood donors, the Bergen Growth Study 2 (19), and anonymous surplus serum samples were obtained from small children visiting the Helse Bergen Trauma Clinic and their Pediatric Cancer Unit. Serum from healthy school children who later got cancer and matched controls were obtained from the UK ALSPAC (14901 donors) and BiB cohorts (20, 21). Study data were collected and managed using REDCap electronic data capture tools hosted at the University of Bristol, a secure, web-based software platform designed to support data capture for research studies (22). At age 18, study children were sent ‘fair processing’ materials describing ALSPAC’s intended use of their health and administrative records and were given clear means to consent or object via a written form. For these cases, we have incomplete information on histological diagnoses.

All patients provided informed consent to participate according to ethical approval 17866 from the Regional Committee for Medical Ethics, which also approved amendments for the inclusion of anonymous samples from healthy controls and additional samples from pediatric patients from the General Cancer Biobank and Norwegian Childhood Cancer Biobank (23), both at Oslo University Hospital, as part of the same approval number.

### Proteome microarray analysis

HuProt v4.0 Human Proteome Microarrays (CDI Labs) were purchased from Cambridge Protein Arrays Ltd. Probing of the arrays and statistical data analyses were performed according to the manufacturer’s protocol. Specifically, the arrays were blocked with 2% BSA at 4 °C overnight. Serum samples were diluted 1:1000 in blocking buffer and incubated with the arrays with gentle shaking for 2h at RT. The arrays were washed 3 times in rapid succession and 2 times for 5 min. The arrays were then incubated with an anti-GST antibody (Cat. no ab117497, Abcam) and anti-human IgG secondary antibodies (Cat. no A-21089, Invitrogen) for 2h at RT, followed by washing as described previously. Detection was performed using the GenePix scanner, with the same settings maintained throughout the entire study. Preprocessing of raw data and statistical analysis were performed according to the standard protocol provided by Cambridge Protein Arrays Ltd. For neighborhood background correction, the net intensities were calculated by subtracting the median local background intensities from the median raw intensities. To obtain the corrected net intensities, a negative control array (no serum) was produced for each batch, and the net intensities were recalculated by subtracting the corrected background intensities from the net intensities for each corresponding spot on the serum-probed array from the same batch. The relative standard deviation (rsd) was calculated for each duplicate spot. For z-score-positive proteins, the cutoff was set at >2.51, corresponding to a p-value of 0.02; for rsd, the cutoff was set at <0.35; and for the sample-to-control ratio (Prot s/c), the cutoff was set at >3.5, as recommended. The significant proteins were ranked using z-scores.

An intrinsic technical drawback of protein array technology is the so-called “batch effect”. We observed that some batches of arrays failed to detect antigens that were previously efficiently detected by other batches. Proteins that were observed to be affected by batch effects were removed from the analysis and disregarded in this study.

### Statistical analysis

For pre-diagnostic cohort, we examined antigen-specific immune responses in cases and controls using generalized linear mixed models. Six positive antigens were analyzed individually (ADRM1, DDX58, DHX58, DMC1, MGMT, and UNG) using a two-stage analytical strategy. First, for each antigen, we fitted a standard logistic regression model to obtain an initial estimate of the association between disease status and antigen reactivity, expressed as odds ratios (ORs).

Second, because multiple blood samples were available from some individuals, we fitted mixed-effects logistic regression models to account for within-individual correlation. These models included a random intercept, allowing individual-specific baseline probabilities of antigen reactivity and thereby reducing bias arising from repeated measurements. To control for multiple testing across the six antigen-specific analyses, p-values were adjusted using the Benjamini–Hochberg false discovery rate (FDR) procedure.

To further assess differences between cases and controls at the group level, we conducted a pooled analysis of the six core antigens. A composite binary variable was created to indicate the presence of at least one core antigen. Individuals with multiple blood samples were classified as positive if any core antigen was detected in any sample and negative otherwise. The association between pooled core antigen positivity and disease status was then evaluated using binary logistic regression, with odds ratios and 95% confidence intervals reported.

The discovery cohort lacked childhood controls, which would allow false positives among pediatric patients for autoantibodies common in healthy children, but this would not be the case in the full study (**Table 1**) where we had obtained a large number of samples from healthy children. In fact, in subsequent stages of our study, the analysis of controls from 0.6 to 19 years of age and of age-matched patients allowed the adjustment of the pediatric cohort for false positives and fully confirmed the same patterns we observed at the discovery stage.

**Table 1:** Heat map showing the frequencies (%) of the most enriched autoantigens in cancer patients and healthy controls.

	Pediatric patients							Adult patients						Healthy controls		
	total 84							total 91						total 141		
N of samples	5	10	10	11	11	16	21		13	12	38	12	16		60	81
Tumor type	Enbryonic	NBL	Wilm's	Glioma	ALL	RMS	OS		ALL	RMS	OS	UPS	LPS		Young	Adult
ALKBH7	20		20	18	9				8						2	1
APBB1		10	30	27	9	13	5		8		3				5	2
DMC1			20		18	19	10								2	1
MGMT	20				9		5		38	42		8			5	5
NUMA1	40	20	10	18	9	6			8		3	8			2	
RAD52	20	40	30	36	36	31	14		23	8					3	
UNG				9		25	5		62	58					10	1
DDX58	20	20	30	18	55	19	10		8		3	17			5	2
DHX58		20	30	18	64	13					5				3	

Colored cells are shaded by prevalence. This core list includes autoantigens that were positive in at least 10% of patients in at least two different cancer groups.NBL, neuroblastoma; ALL, Acute lymphoblastic leukemia; RMS, rhabdomyosarcoma; OS, osteosarcoma; UPS, Undifferentiated pleomorphic sarcoma; LPS, Liposarcoma.

### Cell lines

The panel of osteosarcoma cell lines was either established in-house from patient-derived xenografts (PDXes) from patient tumors (NRH-OS2 and NRH-OS3) or older cell lines (U2OS – RRID: CVCL_0042, SJSA-1 - RRID: CVCL_1697, IOR/OS18 - CVCL_9993, IOR/OS15 -CVCL_9991) available from the STR-validated EuroBoNeT collection (24), BJ (RRID: CVCL_3653) and MRC5 (RRID: CVCL_0440) primary human fibroblasts were obtained from ATCC (RRID: SCR_001672); primary MSC were produced in-house, and NHOst primary human osteoblasts (cat. no CC-2538) were obtained from Lonza, Walkersville, MD, US.

### Primary antibodies

The following antibodies were used for confocal microscopy: ADRM1 (RRID: AB_10794044), APBB1 (RRID: AB_2882385), CGGBP1 (RRID: AB_2079283, RRID: AB_2675433), CRAT (RRID: AB_1847212), DHX58 (RRID: AB_1847665), EEPD1 (RRID: AB_2682223), FBL (RRID: AB_2105788), Golgin-97 (CDF4) (RRID: AB_221447, RRID: AB_2115315), GST (RRID: AB_10900368), HNRNPC (RRID: AB_2117500), Hsc7 (RRID: AB_2120153), anti-Human IgG (Invitrogen cat. no A-21089, RRID: AB_2535745) ING1 (RRID: AB_2681876), INO80E (RRID: AB_10794923), Nucleolin (ZN004) (RRID: AB_2533426), NuMA (RRID: AB_2154616), PLCD4 (RRID: AB_2675855), RSL1D1 (RRID: AB_2678504), RAD52 (Atlas Antibodies, cat. no HPA028957, RRID: AB_2672842), SON (RRID: AB_2674029), SRP19 (Proteintech cat. no 16033-1, RRID: AB_11041714), ZFP36L1 (Proteintech cat. no 12306-1, RRID: AB_2918780), ZFP36L1/2 (RRID: AB_2737443), and for ZO-1 (RRID: AB_2533456).

### Confocal microscopy

The cells were seeded on coverslips in 12-well plates in regular culture medium. The next day, the medium was replaced with fresh culture medium containing donor serum at a 1:40 dilution. The cells were then incubated at 37 °C for 2h. After incubation, the cells were washed twice with PBS, fixed with 4% formaldehyde solution for 15 min, washed twice with PBS, permeabilized with 0.02% Tween-100 solution for 10 min, washed twice with PBS, and blocked for 30 min with 2% BSA solution. The cells were then incubated with anti-human IgG antibody overnight at 4 °C. The next day, the cells were either incubated for 2h at room temperature with primary antibodies against the protein of interest, followed by secondary antibodies, or incubated for 30 min with phalloidin. Finally, the coverslips were mounted on slides with Vectashield mounting medium containing DAPI. The samples were analyzed on Leica SP8-STED confocal microscope at 100X magnification using the basic setting, lightning mode, and z-stack mode for 3D reconstruction.

The serum dilution 1:40 is a standard used in other studies on penetrating autoantibodies. However, we tested a range of dilutions below and above 1:40 to establish the best experimental protocol and concluded that 1:40 indeed was the optimal one. This concentration gives clear signal from internalized IgG with low background and saves scarce serum samples.

### Flow cytometry

Cell Cycle analysis strategy: after exclusion of debris, single cells were gated for the IgG^+^ and IgG^-^ cell population, which was analyzed separately across the samples via the in-built cell cycle analysis tool in FlowJo. Due to biological variability between control serum-treated and patient serum-treated samples, the proportion of IgG^+^ cells differed across conditions, with control serum samples consistently showing a lower percentage of IgG^+^ cells. Each subset (IgG^+^/IgG^-^) in both groups was redefined as 100% to allow calculation of the relative distribution of cells across G0/G1, S, and G2/M phases within that specific fraction. Importantly, this normalization reflects the internal cell cycle composition of each IgG-defined subpopulation and does not represent the proportion of the total sample.

### TISa pattern quantification

The .vsi files from the Olympus VS120 S6 slide scanner acquired with the 10x UPlanSApo NA 0.4 objective were analyzed using QuPath software for quantitative image analysis (version 0.5.1) (25). First, DAPI-stained nuclei were detected with “Cell Detection”, and a radius of the investigated area (ROI) of 10 µm was allowed around the nuclei. Thereafter, “subcellular spot detection” was performed to identify spots with anti-human IgG staining within the ROI. The spot size was defined as an expected size of 50 µm^2^, a minimum spot size of 30 µm^2^ and a maximum spot size of 250 µm^2^. The range was optimized to minimize the number of false-positive and false-negative results. The results of the analysis were confirmed by visual inspection.

### IgG isolation

IgG were purified from the blood serum using the Nab Protein G Spin Kit according to the manufacturer’s protocol (Thermo Fisher Scientific Inc.).

### Availability of data and materials

Requests for materials will be considered. The complete dataset was deposited in the ArrayExpress repository (RRID: SCR_002964) with the accession number E-MTAB-13995. ALSPAC data are available through a fully searchable data dictionary and variable search tool http://www.bristol.ac.uk/alspac/researchers/our-data/. Data Access Statement: Research data supporting this publication are available from the ArrayExpress repository with the accession number E-MTAB-13995 (Public only after publication, use Link for anonymous access).

## Results

### Sera from pediatric cancer patients are enriched in IgG against DNA repair proteins

Osteosarcoma is more common in children and young people, with an incidence that peaks at approximately 19 years of life and then flattens towards older ages (**Figure 1A**). Our discovery cohort consisted of 60 osteosarcoma patients aged between 2 and 76 years at diagnosis and 55 healthy controls aged between 22 and 69 years. Serum samples were screened using high-density proteome arrays to determine the repertoire of circulating IgG autoantibodies.

**Figure 1:**
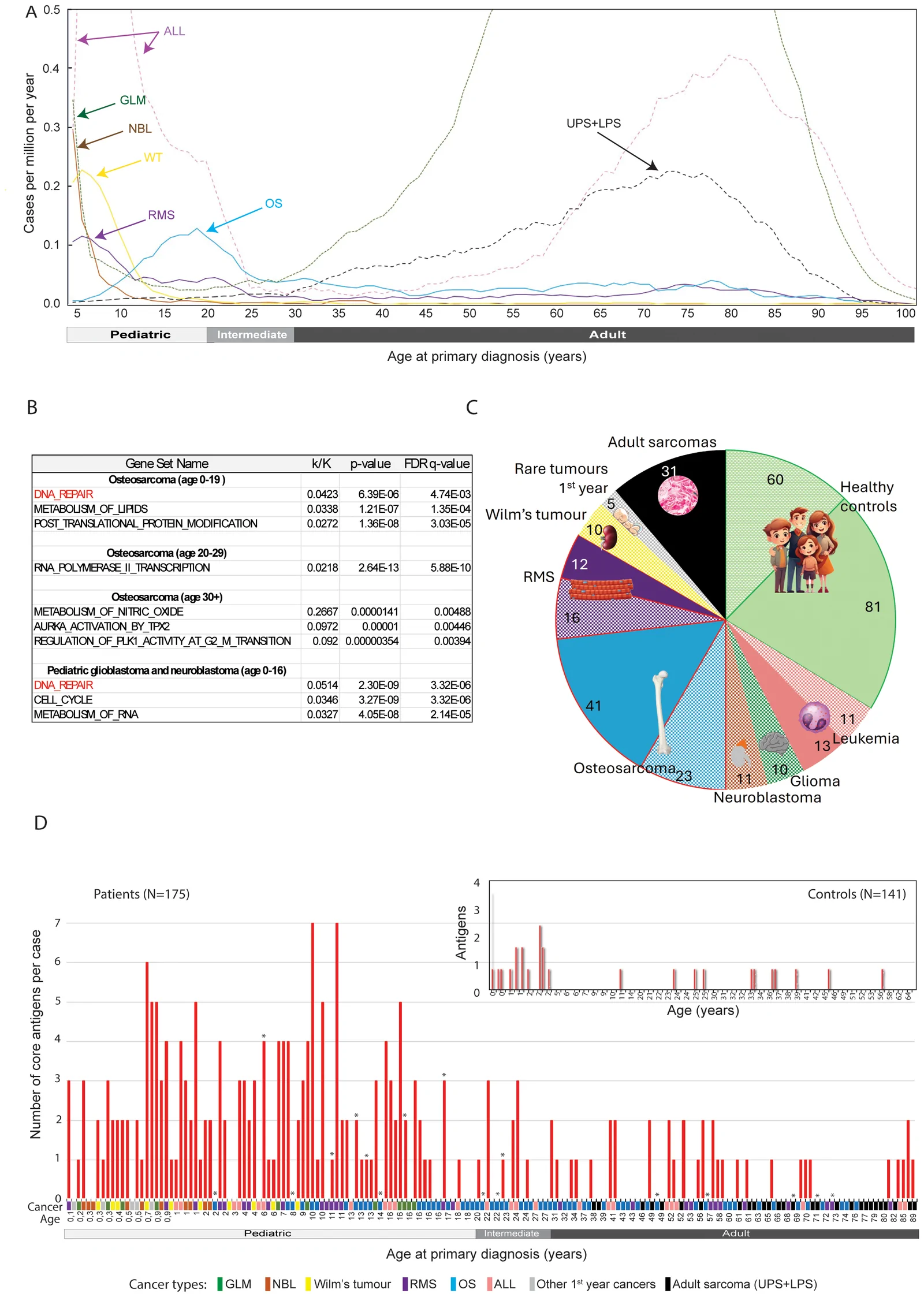
Age distribution of pediatric cancer patients and autoantibodies. **(A)** Age incidence of the cancers included in this study in Norway from 1980 to 2019. The data are plotted as 5-year moving averages for better visibility. The colored arrows indicate the peaks of incidence in children. The bar indicates the pediatric (0-19), intermediate (20-29), and adult (30+) age groups. ALL – acute lymphoblastic leukemia; GLM – glioma; NBL – neuroblastoma; RMS – rhabdomyosarcoma; OS – osteosarcoma; WT – Wilms tumor; UPS – undifferentiated pleomorphic sarcoma; LPS – liposarcoma. Data courtesy of the Norwegian Cancer Registry. **(B)** The three most enriched (highest k/K ratio) molecular pathways for the autoantigen sets (z score > 2.5) from patient groups by GSEA. Full GSEA data are shown in **Supplementary Data Set 1**. **(C)** Graphical presentation of the analyzed cohorts, colored by cancer types as in **(D)**, with healthy controls in green. Adult fractions (above 20 years) in solid color, children and young fractions in dotted shading. **(D)** Number and distribution of autoantigens according to age. Each bar shows the total number of autoantigens per patient from the three categories of our core list (**Table 1**). The cases are sorted by age at diagnosis in ascending order, and the controls are sorted by sampling age. The small squares below the x-axis indicate each case, colored according to cancer type, as shown in the legend, and the asterisks indicate samples taken after initial chemotherapy.

After filtering out autoantibodies shared by patients and healthy controls, we divided the osteosarcoma patients into three age groups, namely, ≤19 (pediatric), 20-29 (young adults), and ≥30 (adult), and subjected the autoantigens detected in each group to gene set enrichment analysis (GSEA) (26). Strikingly, a cluster of DNA repair pathways was among the most significantly enriched in the ≤19 years age group (P = 6.4E-06), whereas there was no significant enrichment in the other two groups (**Figure 1B**). The complete tables of autoantigens in enriched pathways and statistical parameters from GSEA are reported in **Supplementary Data Set 1**.

To determine whether these autoantibody patterns were specific to osteosarcoma or had more general significance, we analyzed additional cohorts of pediatric patients with glioma, neuroblastoma, Wilms tumor, and some rare cancers in the first year of life, as well as two additional age-spanning cohorts of patients with rhabdomyosarcoma (RMS) and acute lymphoblastic leukemia (ALL), plus two adult types of sarcomas, undifferentiated pleomorphic sarcoma (UPS) and dedifferentiated liposarcoma (LPS) (**Figure 1C**; **Supplementary Table S1**). Moreover, we extended our healthy control cohort by adding 37 samples from children younger than 14 years of age. GSEA of the autoantigens that were shared between the pediatric cancer groups confirmed enrichment of the DNA repair pathway (P = 2.0E-04) (**Figure 1B**; **Supplementary Data Set S1**), whereas there was no such enrichment in the adults or healthy children. For a more exhaustive analysis, we carried out a manual functional classification of all detected autoantigens on the basis of information from the Human Protein Atlas (27), GeneCards, the MD Anderson list of DNA repair genes (28), and PubMed, which confirmed GSEA results. Among autoantigens that were most enriched and shared between pediatric patients were RAD52, a pivotal component in transcription-associated homologous recombination repair; NUMA1, a key protein of the mitotic spindle (29, 30); ALKBH7 and APBB1, required for induced programmed necrosis and apoptosis, respectively, in response to DNA damage. The other two DNA repair autoantigens, UNG and MGMT, were strongly enriched in adult and pediatric patients with RMS and in adult patients with ALL.

In addition to anti-DNA repair proteins, pediatric cancer patients with different cancer types were positive for autoantibodies targeting DDX58 and DHX58, involved in innate immunity and antiviral response.

The frequencies of autoantibodies to proteins in the enriched pathways varied between cancer types and ages. For inclusion in our core list (**Table 1**), we selected autoantigens that were positive in at least 10% of patients in at least two different cancer groups. The distribution of these autoantigens across patients’ ages is shown in **Figure 1D** and the distribution per individual with age at diagnosis is given in **Supplementary Data Set S2**.

Although these pathways were not enriched in most adults, some autoantigens were observed at lower frequencies in adult patients, mostly those younger than 30 years of age. Interestingly, among adult patients, leukemia patients stood out for having a profile resembling that of pediatric leukemia patients. Adult RMS patients, on the other hand, had high prevalence of MGMT and UNG autoantibodies, but were otherwise in line with other adult cancer profiles. Since autoantibodies can be induced by tumor neoantigens, we compared our lists of autoantigens enriched in pediatric patients with public tumor mutation data (31–33), including the St Jude Pediatric Cancer Knowledge Base, covering 9000 pediatric cancer samples from multiple cohorts (34). In the corresponding tumor types, we found no systematic expression patterns and no significant mutations of these genes (**Supplementary Table S3**). Only two genes were mutated in 1 to 2% of one or two cancer types. Although cancer therapy could contribute to antitumor autoantibodies, there was no indication that this was the case in the sera collected after treatment (marked by asterisks in **Figure 1D**).

### Autoantigens enriched in cancer patients are detected in healthy children years before cancer diagnosis

To assess whether these autoantibodies were present before cancer diagnosis, we obtained from two British biobanks a unique collection of serum samples from 30 healthy children who got cancer years later (before the age of 30), and 29 age-matched controls (20, 21). Most individuals from both groups had multiple blood withdrawals (2 or 3) during school age. Although this cohort is small, it was the best we could find internationally.

We performed a blind screen of this whole cohort on proteome arrays. Strikingly, we found 3 autoantigens, UNG, MGMT and DDX58, enriched in the pre-diagnostic group with the higher prevalence (of 40, 20 and 27 percent, respectively). **Table 2** shows only positive cases (A) and controls (B) with the number of blood samples and the ages of each individual, while full information on this cohort is provided in **Supplementary Table S2**. To have more insight into autoantibody abundance, we compared the mean z-score values between the groups (**Figures 2A–C**). We found that for all three autoantigens those groups that had increased prevalence also had a significantly higher mean z-score (p<0.0001) compared to the controls. As an initial baseline analysis, standard logistic regression was performed for each of the six core antigens to estimate the association between case status and antigen reactivity. We subsequently evaluated all six core antigens (ADRM1, DDX58, DHX58, DMC1, MGMT and UNG) using mixed-effects logistic regression. Because most individuals had multiple blood samples, these models accounted for within-individual correlation. After adjusting for multiple testing across the six antigen-specific analyses using the Benjamini–Hochberg false discovery rate (FDR) procedure, UNG demonstrated a strong and statistically significant association with case status (OR = 6.7; adjusted p < 0.05), DDX58 showed a large effect size (OR = 8.4) although not statistically significant; DHX58 and MGMT also exhibited elevated odds ratios (OR = 3.9 and OR = 3.0, respectively), but this associations were also not statistically significant after FDR correction. The cumulative prevalence of these three autoantibodies was 57% (17 individuals) in cases and 14% (4 individuals) in controls. ADRM1 and DMC1 showed very low prevalence, with no positive observations among controls. ADRM1 was detected in only 1 of 62 case samples and 0 of 58 control samples, while DMC1 was detected in 3 of 62 case samples and 0 of 58 control samples. The effect estimates from the initial standard logistic regression were essentially unchanged compared with those obtained from the mixed-effects models. Therefore, the mixed-effects models are presented as the primary analysis because they account for repeated samples within individuals.

**Table 2:** Data on the positive samples from British cohort.

A							
Case #	# samples	Age at sampling	Age at diagn.	Years to diagn.	DDX58	UNG	MGMT
30	2	9	11	2			
2	2	10	13	3			
6	3	10	14	4			
4	3	8	13	6			
5	3	7	13	6			
8	3	10	17	7			
13	1	12	20	7			
18	3	12	22	10			
19	3	12	22	10			
12	2	7	19	12			
22	2	12	24	12			
24	3	12	25	13			
15	3	8	21	13			
25	2	12	25	14			
17	2	8	21	14			
26	2	12	25	14			
27	3	12	26	15			

B							
Control #	# samples	Age at sampling			DDX58	UNG	MGMT
Ctrl20	2	12					
Ctrl17	2	8					
Ctrl03	3	11					
Ctrl23	3	12					

• N of bl. samples = number of different blood withdrawals for the same person. • Age at sampling = age at the blood withdrawal in samples tested positive for a given autoantibody. For each individual, the number of serum samples (#samples) and age at blood collection (Age at sampling) is shown. For future cases, it is shown age at cancer diagnosis (Age at diagn.) and number of years from blood collection of the positive sample to diagnosis (years to diagn.). The table reports only individuals positive for the autoantigens listed (indicated in red). The information on the full cohort, the cancer types, and positive autoantigens is in **Supplementary Table S2**.

**Figure 2:**
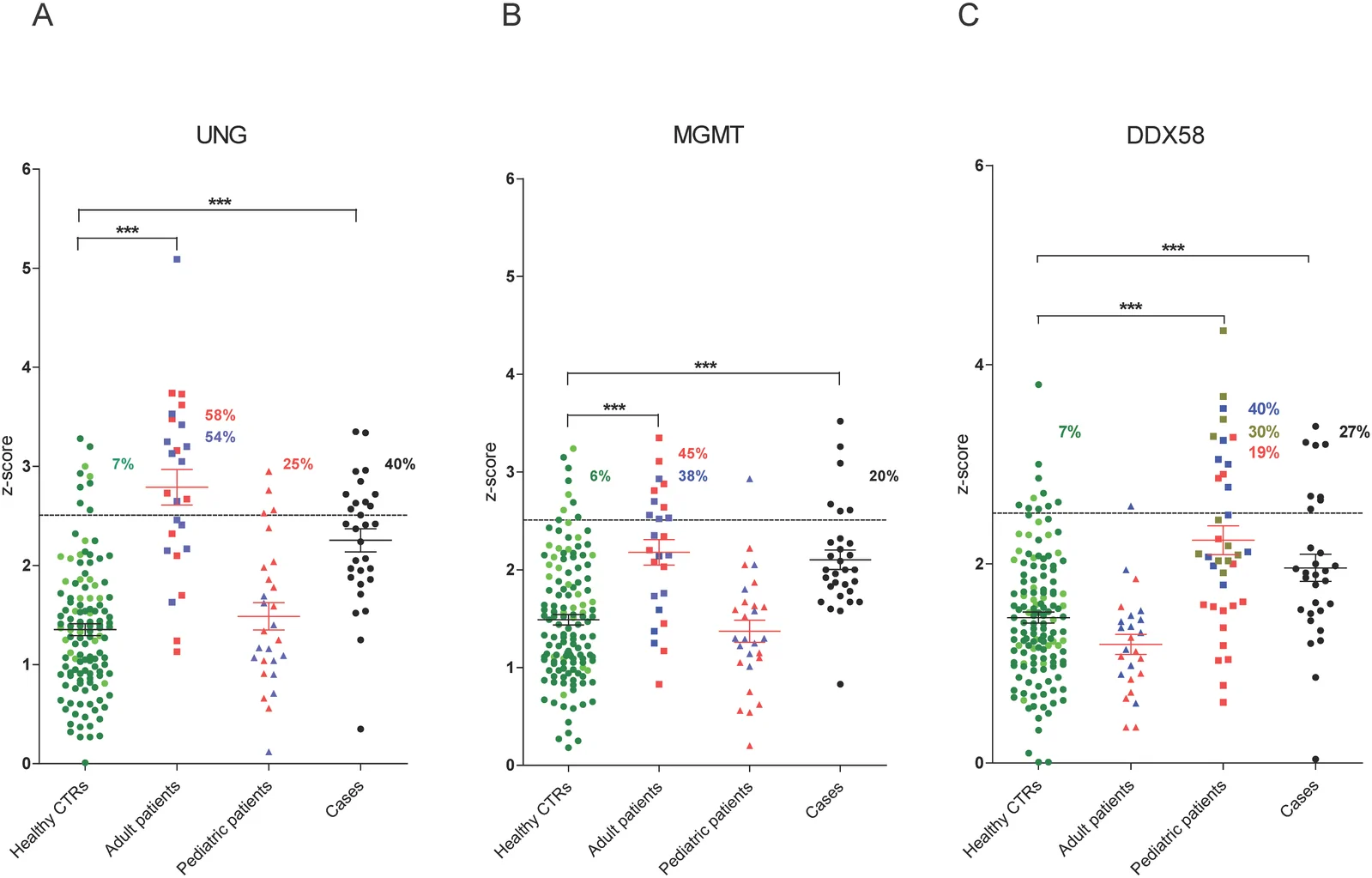
Z-score distribution and prevalence of UNG, MGMT and DDX58 across cohorts. The distributions of z-scores are represented by dot-plots for UNG **(A)**, MGMT **(B)**, DDX58 **(C)**. Each spot shows the z-score for one corresponding sample. The prevalence in different groups is indicated as a % of positive samples (z-score cut off > 2.51 as indicated by the dashed line). The healthy control group includes serum samples collected in Norway (dark green) and the UK (light green) and spans the ages from 6 months to 90 years. In the adult and pediatric patient groups, the color shows the types of cancer: red = RMS, blue = leukemia, brown = Wilms tumor. Cases are children from the British biobanks who were healthy at blood collection but developed cancer between 5 and 15 years later. Error bars = SEM. Three stars indicate p<0.001 relative to the difference between the median of z-score values of the indicated groups.

When all core antigens were pooled into a single composite endpoint, a clear and highly significant difference between cases and controls emerged. Cases had substantially higher odds of having at least one core antigen compared with controls (OR = 8.0; 95% CI: 2.54–24.86; p = 0.0003). This result indicates that children who later got cancer were about eight times more likely than healthy controls to exhibit immune reactivity to at least one core antigen. The pooled analysis captured a strong group-level signal that was not consistently detectable in single-antigen analyses.

Notably, this study included blood samples taken on average 9 years *before* cancer was diagnosed. Unlike our pediatric *patient* group, 50% of UK cases were already adults above 20 years of age at diagnosis. Importantly, their cancer types were not classically pediatric, but mostly adult types and different from cancer types of our main cohort of cancer patients (**Supplementary Table S2**).

### IgG from pediatric patient sera enter cells more efficiently during the G2/M phase

It is well documented that some autoantibodies can penetrate live cells and affect intracellular processes (35–42). The discovery of the peculiar enrichment of autoantibodies in our pediatric patients, and especially, in children before cancer onset, prompted us to consider a potential pathogenic role. This would require the ability of autoantibodies to directly or indirectly inhibit their target antigens, which are intracellular. We used confocal microscopy to investigate the intracellular localization and patterns of IgG from patients and healthy controls in normal and cancer cell lines. Confirming previous reports, we observed penetration of IgG into live intact cells as a general phenomenon present in both patients and healthy controls, regardless of age (**Supplementary Figure S1**).

One variable state in a cell culture that could explain why only a fraction of cells is penetrated by IgG is their cell cycle phase. Flow cytometric analyses revealed that, when SJSA-1 osteosarcoma cells were incubated with sera from pediatric patients, the fraction of IgG^+^ cells was significantly greater in the G2/M phase than in the other phases (**Figure 3A**). Under standard conditions miniaturized due to the limited amounts of patient serum, we did not obtain enough IgG^+^ cells with healthy control serum for cell cycle analysis. To overcome this limitation, we did scaled-up experiments using 10 cm dishes for control samples. The results confirmed that the distribution across cell cycle phases with control serum was the same as with patient serum, although the total fraction of IgG^+^ cells was smaller (**Supplementary Figure S3A**).

**Figure 3:**
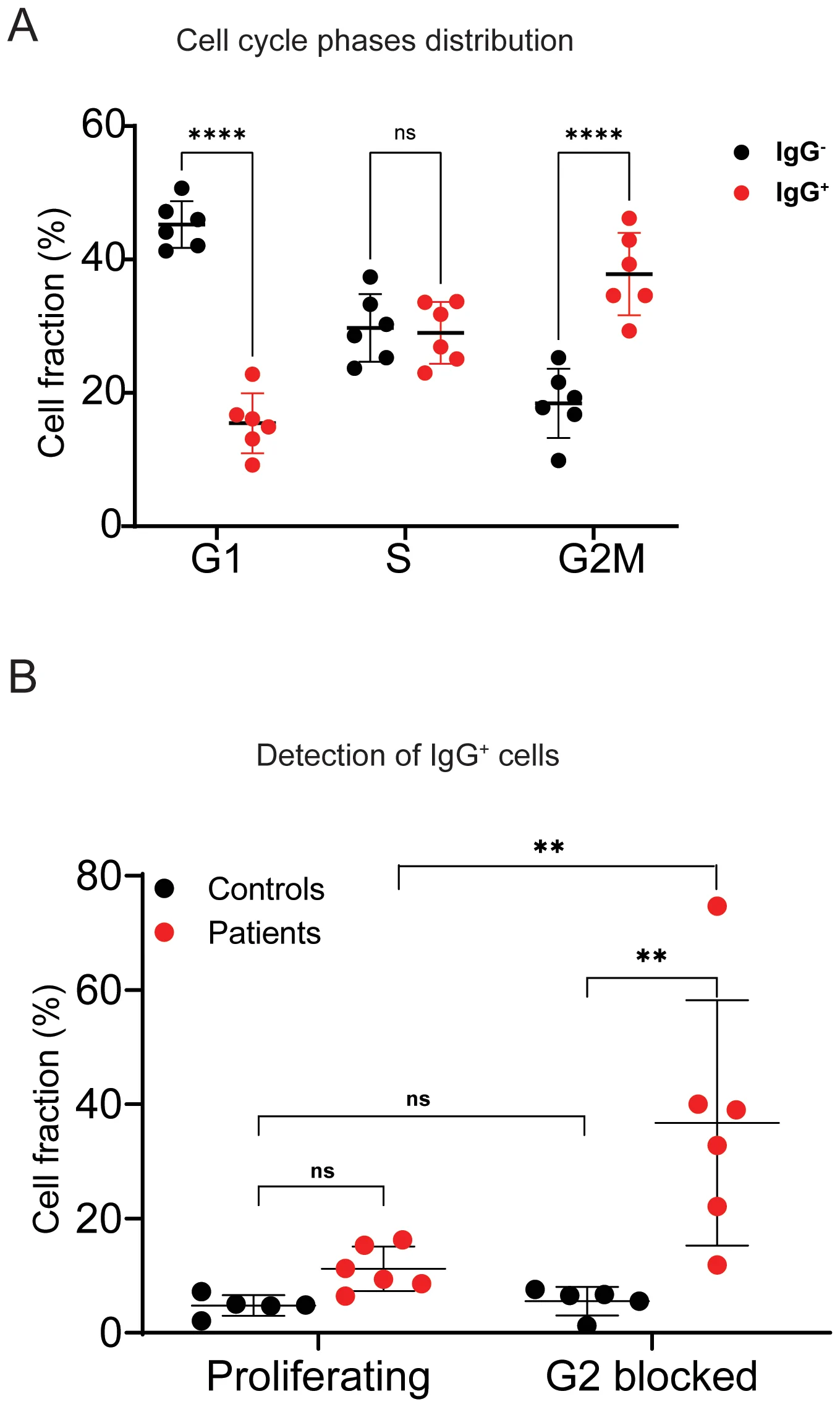
Cell-cycle distribution of IgG^+^ cells. **(A)** SJSA-1 cells were incubated for 2 hours with serum from pediatric patients or controls (N = 6 and N = 5 respectively). Cells were analyzed by flow cytometry and first gated for the IgG^+^ and IgG^-^ cell populations; each population was analyzed separately by the in-built cell cycle analysis tool in FlowJo. Only patients data are shown; controls data are in the **Supplementary Figure S3** **(B)** SJSA-1 cells were blocked with colchicine and incubated either with patient or control serum for 2 hours. Cell-cycle distribution of IgG^+^ or IgG^-^ cells in both cohorts was assessed by flow cytometry as described above. The two stars indicate p-value<0.01; four stars indicate p-value<0.0001.

This observation was corroborated by blocking SJSA-1 cells in G2/M phase by colchicine followed by incubation with patient or control serum. Colchicine-treatment arrested 80% of cells in G2/M (**Supplementary Figure S3B**). Whereas incubation of these cells with patient sera gave 37 ± 21% of IgG^+^ cells versus 11 ± 4% without block, there was no significant difference in healthy control sera, 6 ± 3% versus 5 ± 2% without block (**Figures 3B**; **Supplementary Figure S2C**).

### Penetrating autoantibodies from pediatric patients localize to the TIGER domain

While with IgG from controls, cell-penetration was very sporadic with nonspecific patterns, with IgG from pediatric patients we detected a very specific perinuclear pattern in a larger number of cells (**Supplementary Figures S1A, B**). This perinuclear IgG staining with highly reproducible morphology resembled the TIS granule-endoplasmic reticulum (TIGER) domain, a recently described membraneless organelle associated with the endoplasmic reticulum (ER) (43). We used an antibody against TIS11B (ZFP36L), a protein that builds the scaffold of TIS granules, to locate the organelle. Indeed, the perinuclear IgG formed a “cloud” tightly associated with TIS granules (**Figure 4A**; **Supplementary Figures S1C–G**). We named this IgG pattern TIS-associated (TISa). Next, we determined the localization of TISa with respect to the other two perinuclear organelles, the ER and Golgi. We used calreticulin (CALR) and signal recognition particle 19 (SRP19) as markers for the ER and GOLGA1 for the Golgi. Unexpectedly, the part of the ER occupied by the TISa patterns was not stained by either ER marker but appeared as a “black hole” (**Supplementary Figures S1H–M**). The Golgi, on the other hand, showed a regular localization, distant from the TISa site (**Supplementary Figure S1N**). Interestingly, TISa patterns were present in a variety of sizes and shapes but always had a meshwork structure (**Supplementary Figures S1O–Q**). To confirm that TISa IgG pattern represents a distinct feature of pediatric cancers, we quantified the patterns from the sera of 21 pediatric patients and 21 healthy controls using automated QuPath analysis (24) of fluorescence scanner images with a larger number of cells. We found that 52% of the pediatric patients had a significantly greater fraction of TISa pattern-positive cells (p<0.0001), ranging between 5% and 30% of the total cell population (**Figure 4B**). The TISa pattern was not a feature of only cancer cells, as we detected it with the sera or purified IgG from patients in primary mesenchymal stem cells, normal human osteoblasts, and MRC5 fibroblasts (**Supplementary Figure S1**).

**Figure 4:**
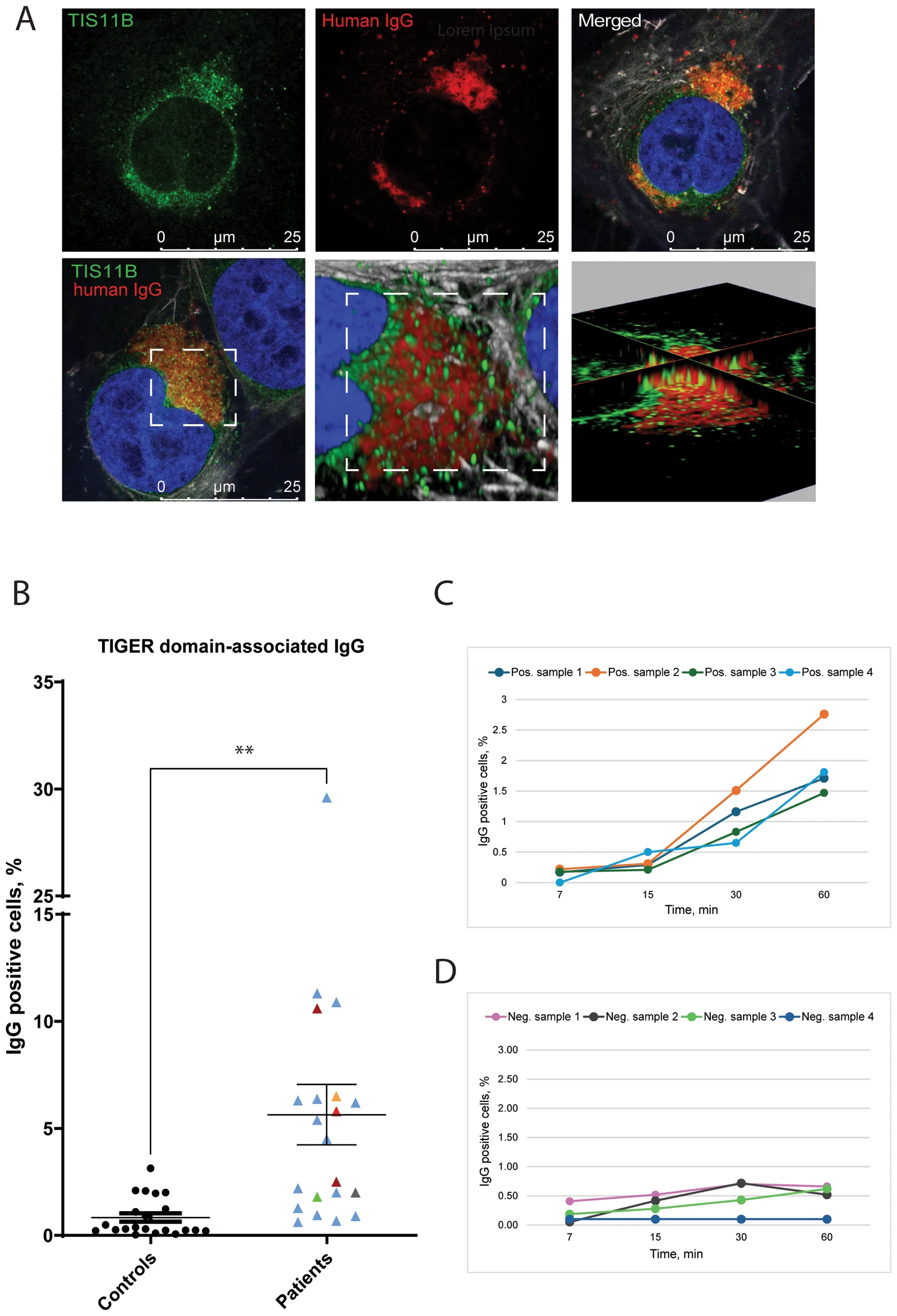
Penetrating IgG from patient serum associate with TIGER domain. SJSA-1 cells were incubated for 2 hours with serum from pediatric patients or controls. **(A)** Detection of penetrating human IgG and TIS granules (top panel); 3D reconstruction of confocal images of the TIGER domain and human IgG (lower panel). SP8 confocal microscope, LIGHTNING mode, 100X magnification. **(B)** Quantitation of cells positive for TIGER-associated IgG from patients or controls following 2 hours of incubation with serum. Perinuclear spots were counted using the QuPath platform. The cases are colored as in **Figure 1** to indicate the cancer type. (three stars indicate p-value <0.01). **(C, D)** Time-course experiment assessing TIGER-association of IgG from positive and negative serum samples in SJSA-1 cells. Cells were incubated for the indicated time-points, up to a maximum of 1 hour. The quantification was performed as in **(B)** at each indicated time-point.

Additionally, to determine the kinetics of TISa accumulation, we carried out a time-course experiment on 4 TISa-positive and 4 TISa-negative serum samples. All the positive samples showed a clear progressive increase of the fraction of TISa-positive cells within one hour of incubation with the sera, whereas the curve in negative samples remained flat (**Figures 4C, D**).

### Penetrating autoantibodies from pediatric patients affect subcellular localization of autoantigens

To assess whether penetrating IgG can target and possibly affect their antigens, we checked their colocalization with 12 proteins randomly selected from our core and extended list of enriched autoantigens. Strikingly, we found that 6 autoantigens, RAD52, DHX58, CRAT, CGGBP1, ING1, and HNRNPC, were sequestered inside TISa “clouds” (**Figure 5**; **Supplementary Figure S2, A-K**), whereas the localization of the autoantigens NUMA1, INO80E, ADRM1, RSL1D1, APBB1, and UIMC1 was not affected. As an additional control, we included in this test 4 ubiquitously expressed proteins that we never detected as autoantigens by protein arrays: SON, FBL, HSC70, and NCL. None of these proteins tested positive for TISa colocalization (data not shown). The 6 positive proteins have different subcellular localizations, as assessed by primary antibodies and consistent with the Human Protein Atlas database: RAD52, HNRNPC, and ING1 are strictly nuclear; CRAT and DHX58 are localized to the Golgi and cytosol; and CGGBP1 is localized in the nucleus and cytosol. However, penetrating IgG did not colocalize with the corresponding autoantigens at these sites.

**Figure 5:**
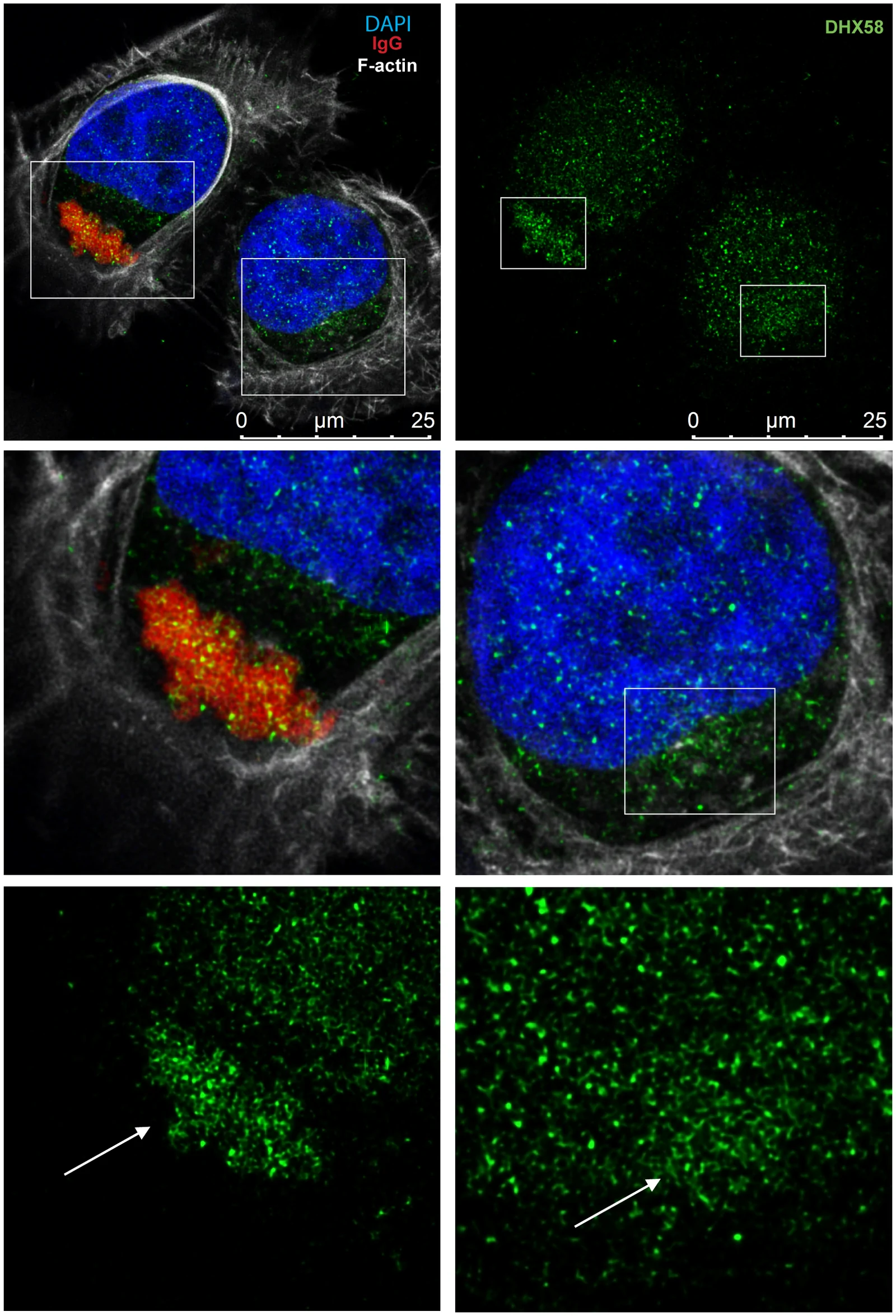
Representative fluorescence images showing autoantigens sequestered inside the “cloud” of the TISa pattern. SJSA-1 cells were incubated with patient serum for 2 hours, fixed and stained. Upper panel shows two representative cells, one with penetrating IgG and one negative. White squares show the DHX58 (green) localized inside the TISa cloud in the positive cell and in Golgi in the negative cell. Middle panel shows a zoomed in view of the areas mentioned above; lower panel shows a magnification of the same areas only with DHX58 staining. The arrows indicate the foci of DHX58 inside the TISa cloud (left) and the regular localization of DHX58 in the Golgi (right). SP8 confocal microscope, LIGHTNING mode, 100X magnification. See also **Supplementary Figures S2A-K** and **Supplementary Videos S1**.

Compared with their regular localization, the proteins sequestered inside TISa often formed larger and brighter foci (**Figure 5**; **Supplementary Figure S2 A-K**). Interestingly, only 14 out of 34 tested patient sera were positive for the colocalization of at least one of these 6 autoantigens in the SJSA-1 cell line.

## Discussion

Pediatric cancers are known to differ fundamentally from adult ones, but the understanding of underlying etiology remains a challenge, even more so now when the incidence of cancer in children and young people is increasing (44, 45). Interestingly, this coincides with an increase in autoimmune diseases (46). The interference of disease-associated penetrating autoantibodies with the activity of cell proteins, both *in vitro* and *in vivo*, is well established (47–49).

Notably, a cloned autoantibody against RAD51 from a patient with lupus was shown to penetrate cells and sequester RAD51 in the cytoplasm, leading to the inhibition of DNA repair (47). The same group recently reported that a patient-derived cell-penetrating anti-topoisomerase 1 autoantibody isolated from a scleroderma patient, inhibited the activity of the protein, leading to cell dysfunction (50). On this background, the repertoire of autoantibodies observed in many of our cancer patients, the penetrating capacity of their IgG and the sequestration of autoantigens in TISa, together provide a plausible mechanism for increased cellular damage that could lead to cancer development, as has already been suggested (51). Although we cannot at this time exclude that the autoantibodies observed could be induced by some underlying preneoplastic process or immune dysfunction, the functional similarity of these target antigens and proteins of which dysfunctional hereditary variants predispose to cancer suggests otherwise. In fact, such penetrating autoantibodies could phenocopy the effects of germline mutations in tumor suppressor genes. The detection of the same autoantibodies found in cancer patients also in healthy children later diagnosed with cancer strongly reinforces the hypothesis of pre-existing autoantibodies that could predispose to the disease. Last, but not least, in the majority of positive pre-diagnostic samples these autoantibodies were detected between 6 to 15 years before diagnosis, which is compatible with the commonly accepted timing of cancer initiation estimated between 5 to 20 years, although we cannot completely exclude that these autoantibodies are very early markers of cancer. Despite this cohort was small and with mostly adult-type cancers, it was enriched with 3 out of 9 of our core autoantibodies. The unusually early incidence of adult-type cancers in so young people suggests some kind of predisposition, which could well be autoimmune predisposition associated with these autoantibodies.

We show for the first time that cell-penetrating autoantibodies from patient sera associate with the recently described TIGER domain and sequester some antigens inside TISa clouds. It is tempting to speculate that the TISa cloud might act as a “sponge” that depletes these proteins from their regular sites of action. Importantly, the functions of TIGER are numerous, it is a hub for processing of certain mRNAs and their products, including short-lived transcription factors. The effects of phase separation on the regulation of cell growth and malignant phenotypes are numerous, and it seems likely that TISa IgG may exert its function through such a mechanism (52–54).

As a multifactorial disorder with complex pathogenesis, cancer can be initiated by an interplay of factors and by their different penetrance, as is the case of mutations. Importantly, we showed that penetrating autoantibodies preferentially enter cells in the G2/M phase, which would be the most vulnerable to interference with DNA damage/repair pathways. Moreover, the presence of multiple autoantibodies targeting the same or related pathways in the same cell could be more detrimental to a cell than a hemizygous mutation affecting a single protein.

Furthermore, many DNA repair proteins are pleiotropic and control multiple critical cellular functions (55). Therefore, interfering with their activities or localization may predispose to cancer by mechanisms other than somatic mutations.

Further research is needed to understand the mechanisms involved in penetration and functional impacts, as well as possible predisposing effects. It could open the door to the development of predictive cancer biomarkers. Ideally, to prove the hypothesis, *in vivo* experiments with specific monoclonal autoantibodies should be done in a cancer-prone mouse model.

## Conclusions

We propose that a preexisting subclinical autoimmune condition characterized by penetrating autoantibodies may compromise genome integrity or other vital cellular functions in critical periods of accelerated tissue growth or differentiation and therefore can predispose to cancer. This model certainly requires final proof but may also lead to new opportunities for cancer prevention.

## Data Availability

Research data supporting this publication are available from the ArrayExpress repository with the accession number E-MTAB-13995.
