# Supplementary material for: Pathogenic autoantibodies targeting DNA repair proteins are shared by cancer patients and healthy children later diagnosed with cancer
Source: Front Immunol. 2026 Oct 14;17:1911932. doi: 10.3389/fimmu.2026.1911932 (PMC13713722; doi:10.3389/fimmu.2026.1911932)
Supplement: Supplementary file 1 [file DataSheet1.zip › Supplementary Data/Supplementary Figure S1-S2.pdf]

Supplementary figure S1

(A-G) TIGER domain and
TISa pattern detection

Patient ID OS18

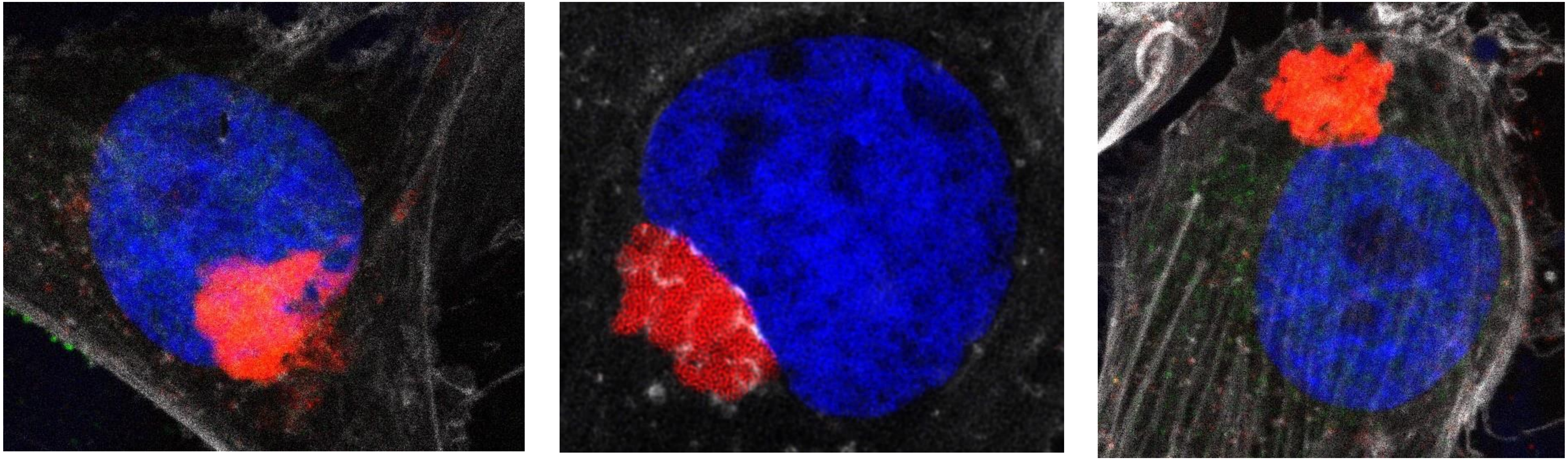

Normal human osteoblasts, fixed, permeabilized and stained with anti-human IgG
DAPI, blue
Anti-human IgG, red
Phalloidin, grey
Confocal microscope, 100X

Representative pictures

A

Patient ID OS18

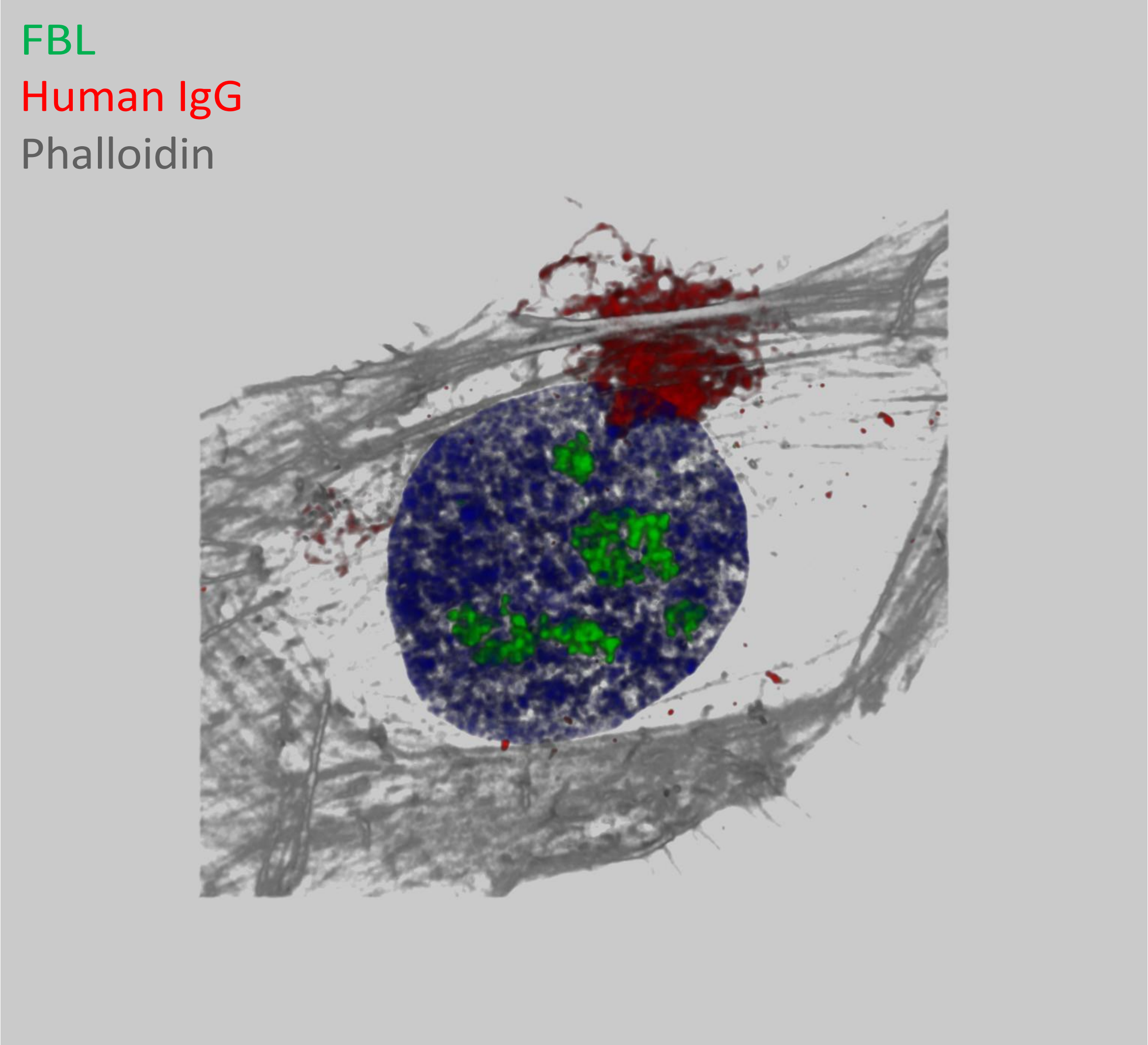

SJSA-1

3D reconstruction of z-stack mode
Confocal microscope 100X

B

Patient ID OS28

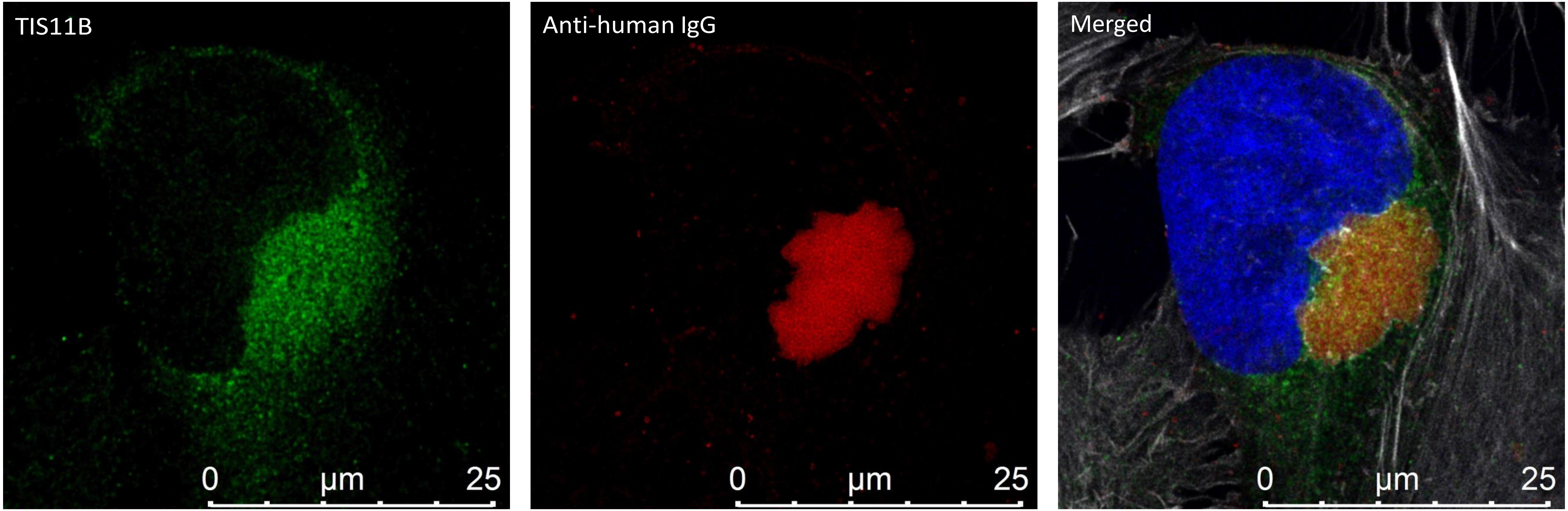

SJSA-1
DAPI, blue
Anti-human IgG, red
Anti-TIS11B, green
Phalloidin, grey
Confocal microscope, 100X

C

Patient ID OS28

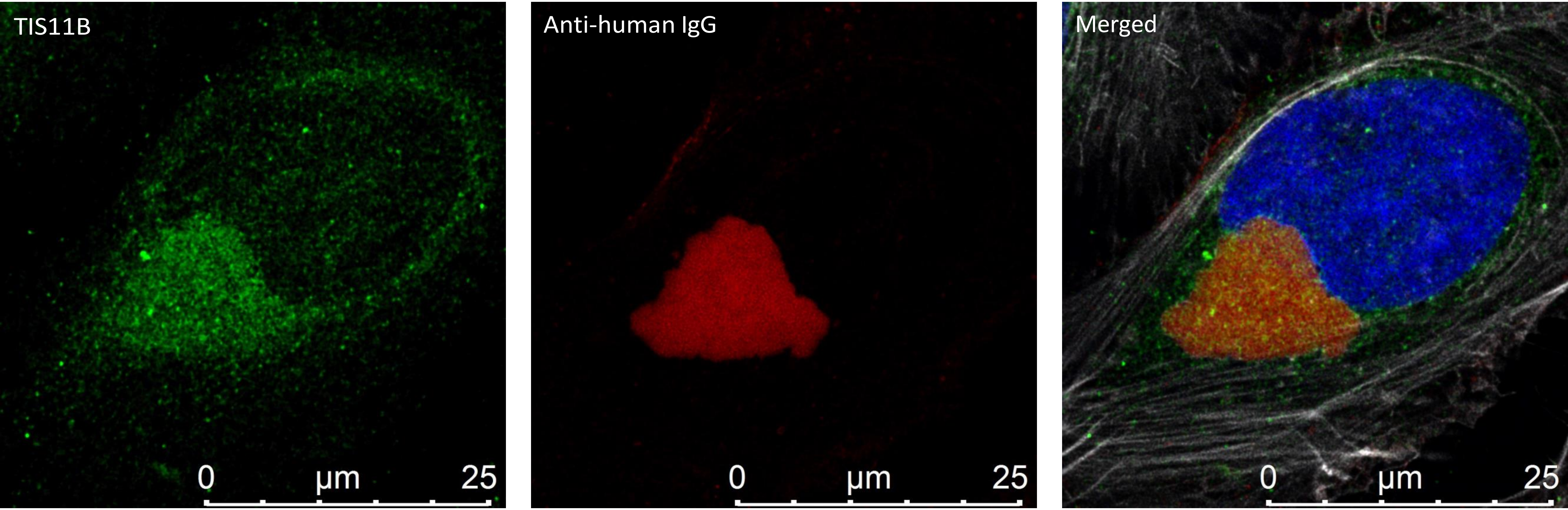

SJSA-1
DAPI, blue
Anti-human IgG, red
Anti-TIS11B, green
Phalloidin, grey
Confocal microscope, 100X

D

Patient ID OS62

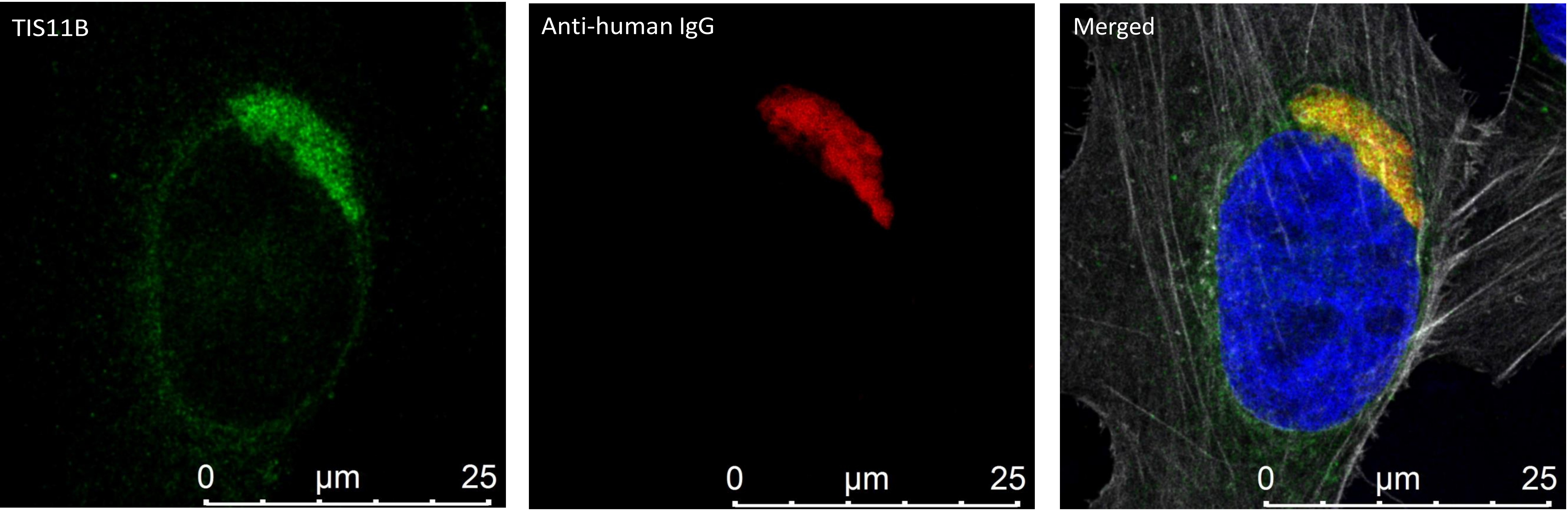

SJSA-1
DAPI, blue
Anti-human IgG, red
Anti-TIS11B, green
Phalloidin, grey
Confocal microscope, 100X

E

Patient ID OS8

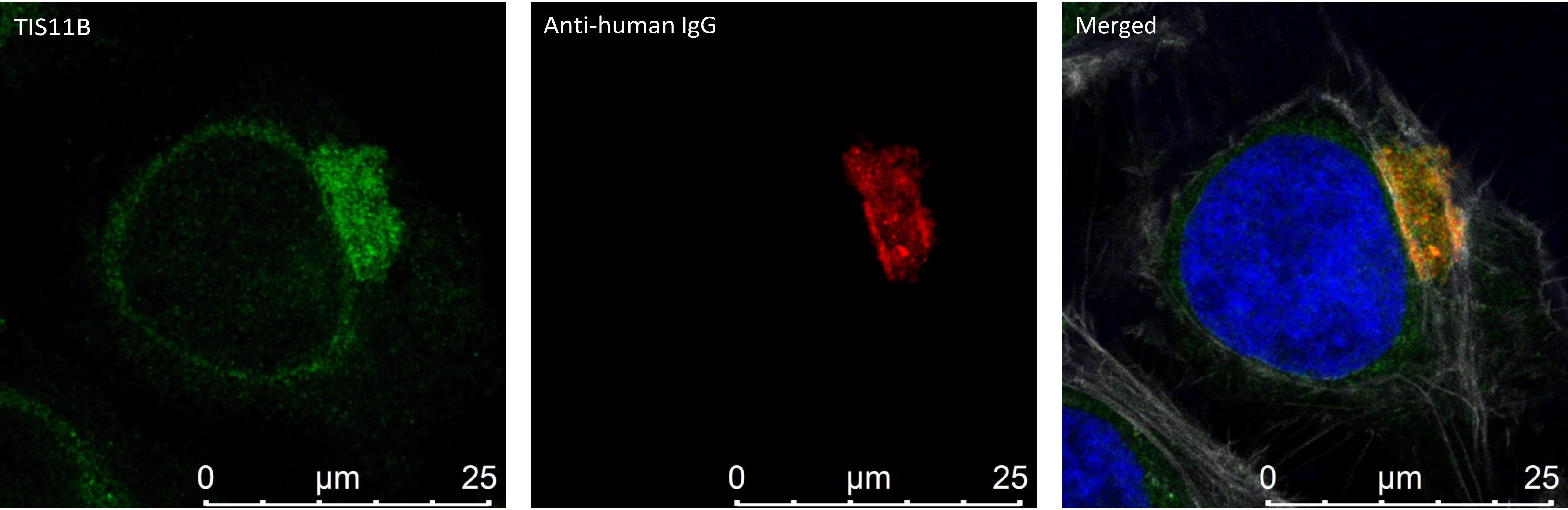

SJSA-1
DAPI, blue
Anti-human IgG, red
Anti-TIS11B, green
Phalloidin, grey
Confocal microscope, 100X

F

Patient ID OS8

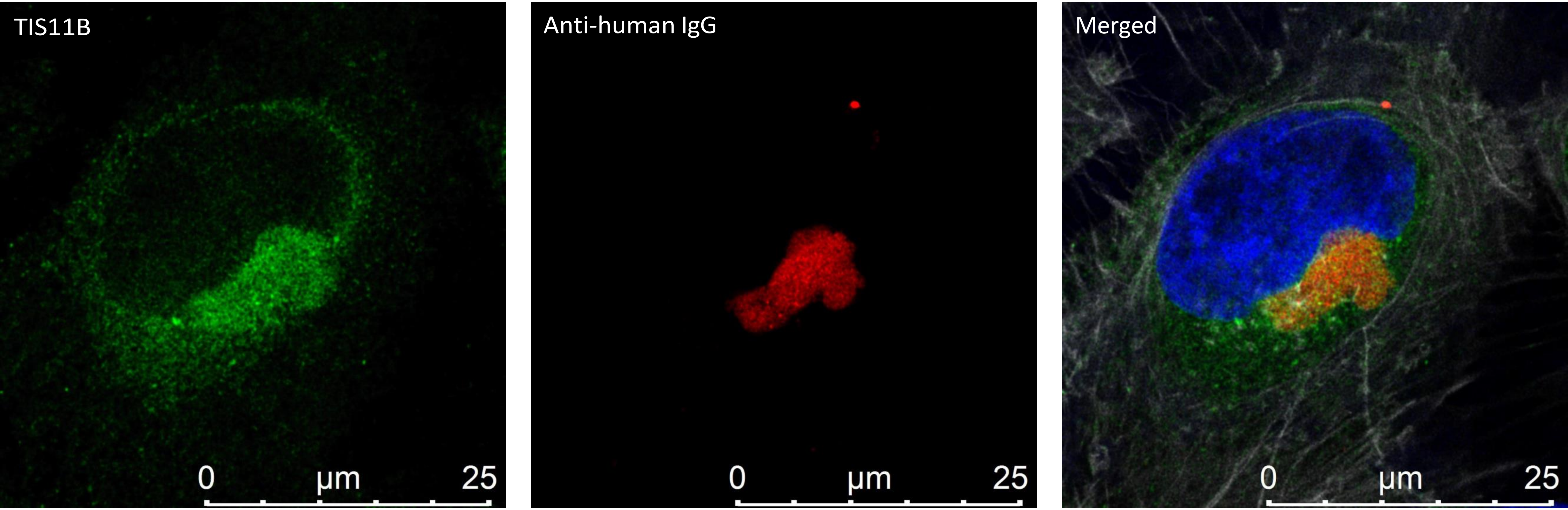

SJSA-1
DAPI, blue
Anti-human IgG, red
Anti-TIS11B, green
Phalloidin, grey
Confocal microscope, 100X

G

Supplementary figure S1

(H-J) CALR (Calreticulin) with serum from pediatric patients

Rabbit polyclonal antibody, Proteintech

Patient ID AS1 (AngioSarcoma)

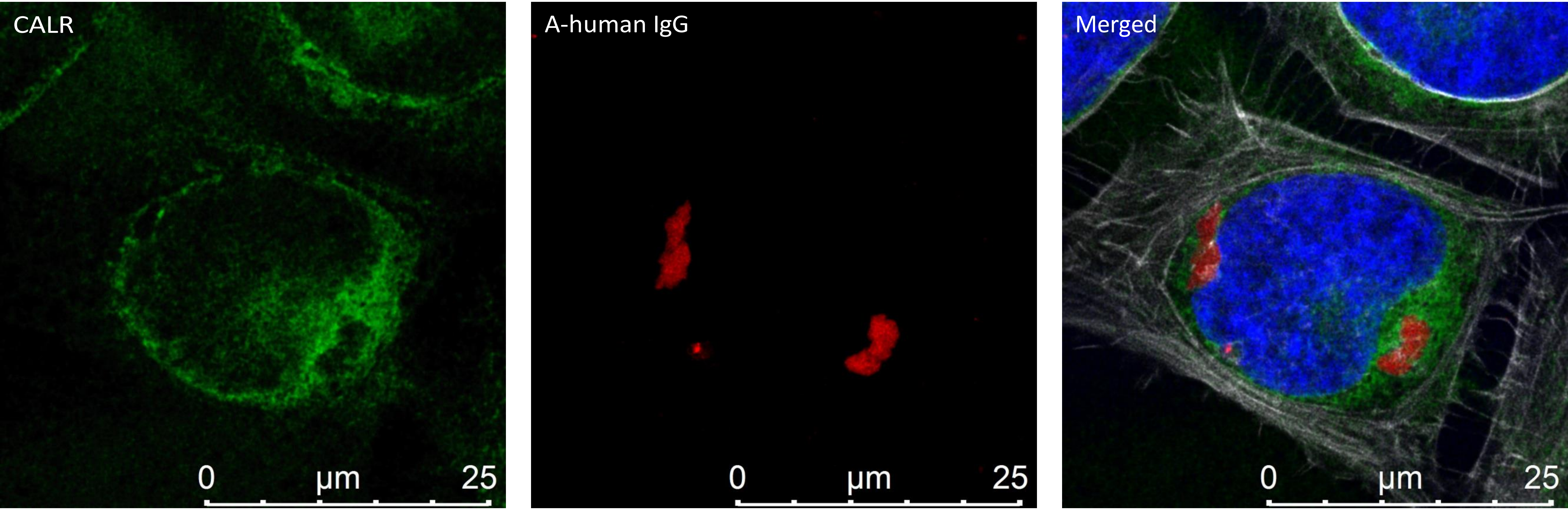

SJSA-1
DAPI, blue
Anti-human IgG, red
Anti-CALR, green
Phalloidin, grey
Confocal microscope, 100X

H

Patient ID AS1 (AngioSarcoma)

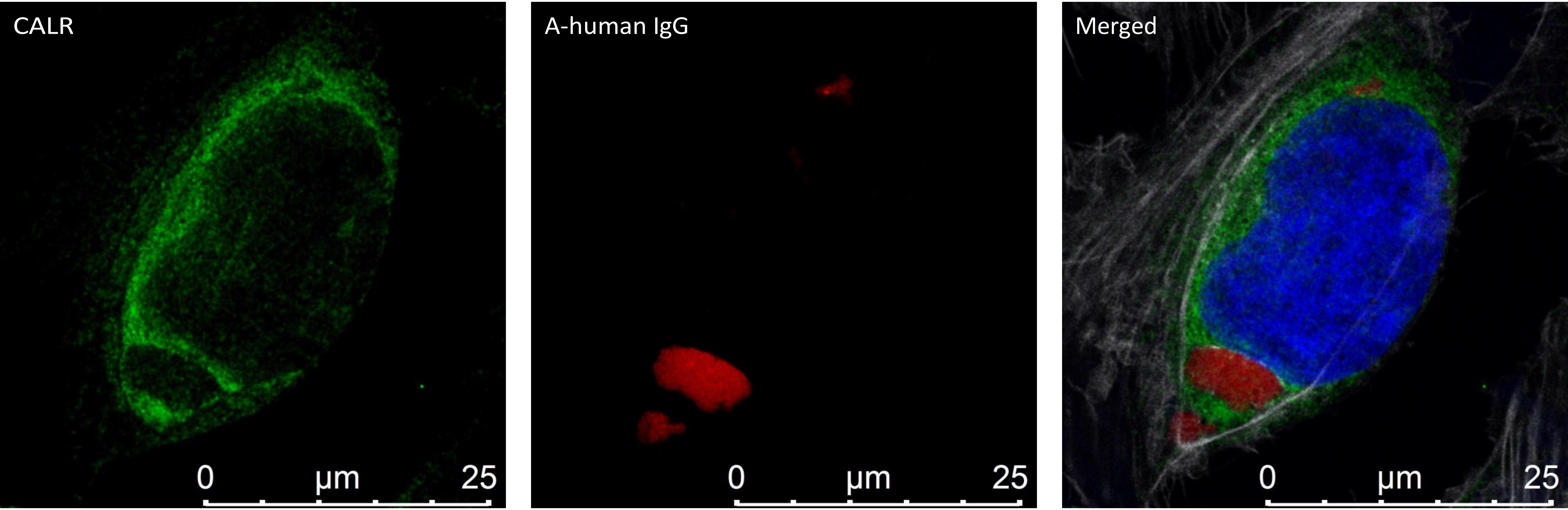

SJSA-1
DAPI, blue
Anti-human IgG, red
Anti-CALR, green
Phalloidin, grey
Confocal microscope, 100X

Patient ID AS1 (AngioSarcoma)

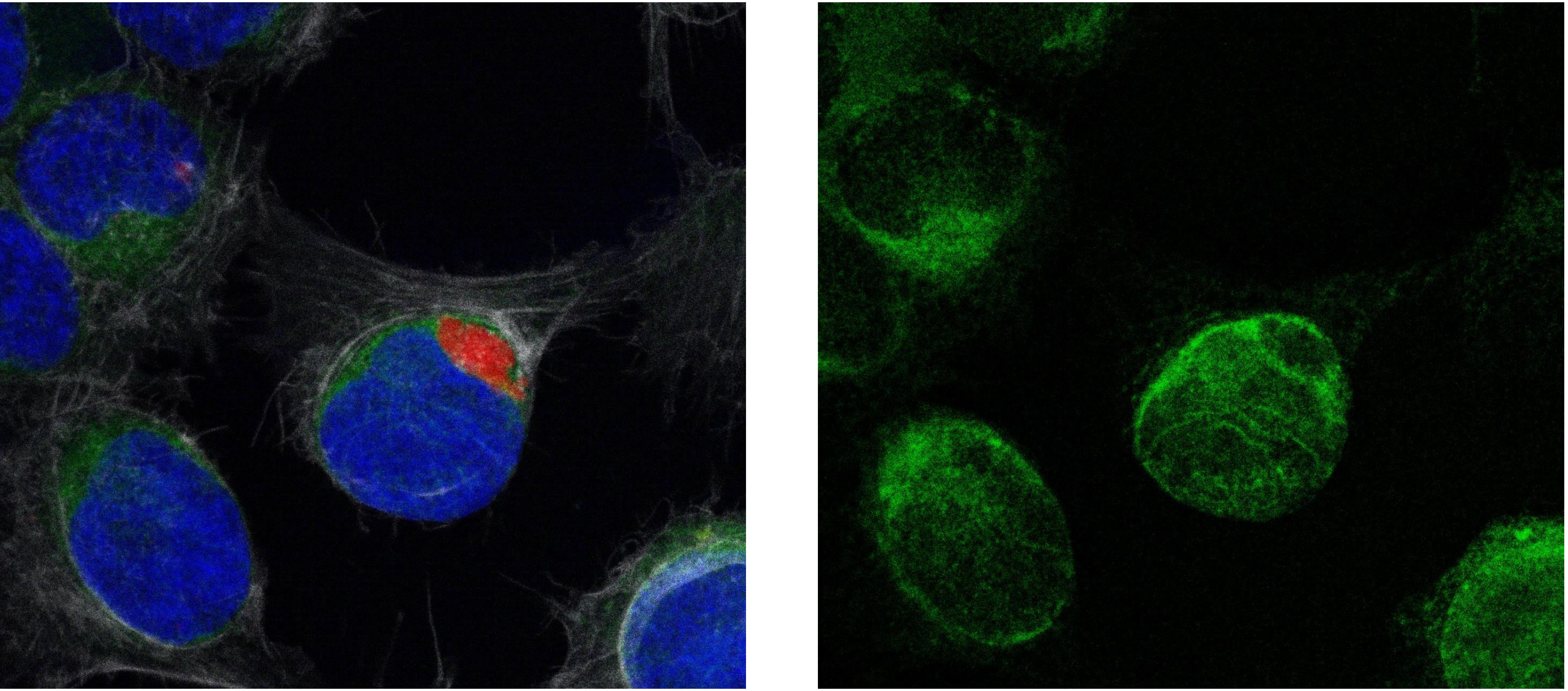

SJSA-1
DAPI, blue
Anti-human IgG, red
Anti-CALR, green
Phalloidin, grey
Confocal microscope, 100X

J

Supplementary figure S1

(K-M) SRP19 with serum from pediatric patients

Rabbit polyclonal antibody, Proteintech

Patient ID OS15

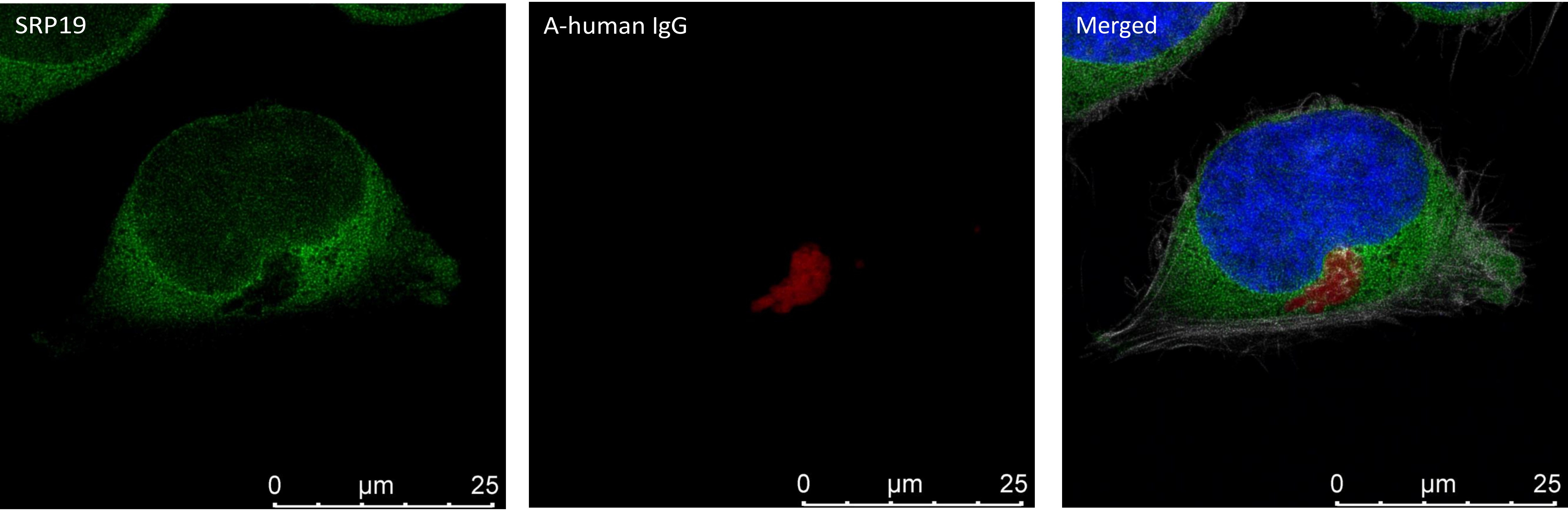

SJSA-1
DAPI, blue
Anti-human IgG, red
Anti-SRP19, green
Phalloidin, grey
Confocal microscope, 100X

K

Patient ID OS15

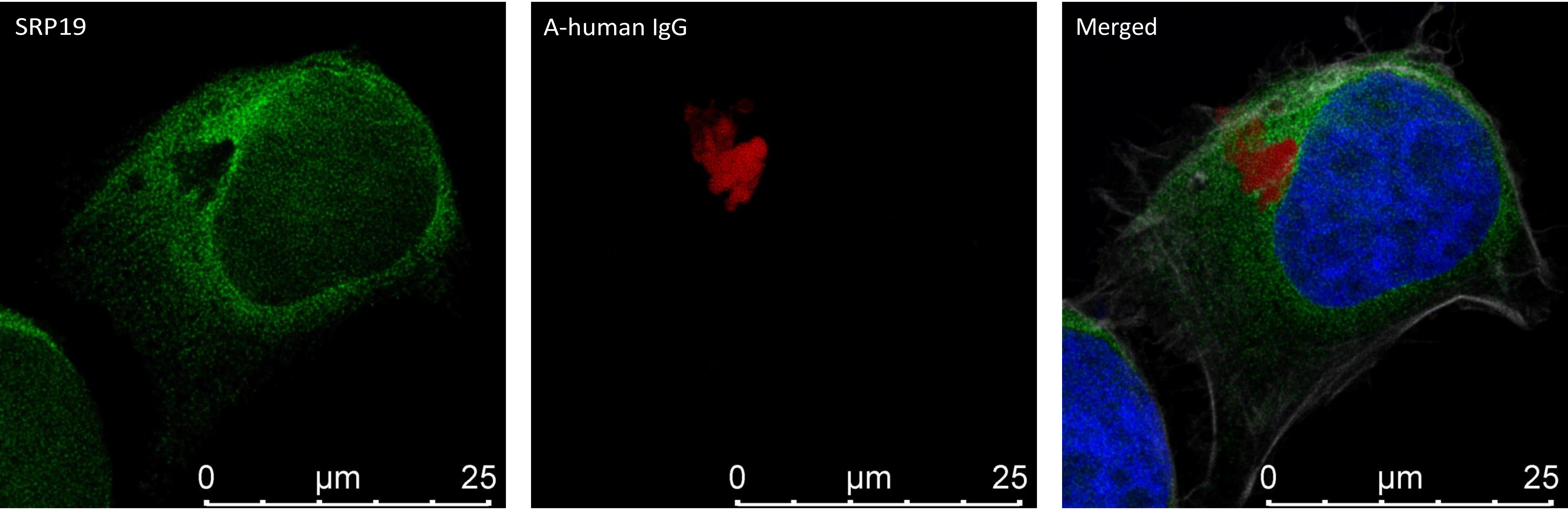

SJSA-1
DAPI, blue
Anti-human IgG, red
Anti-SRP19, green
Phalloidin, grey
Confocal microscope, 100X

L

Patient ID OS15

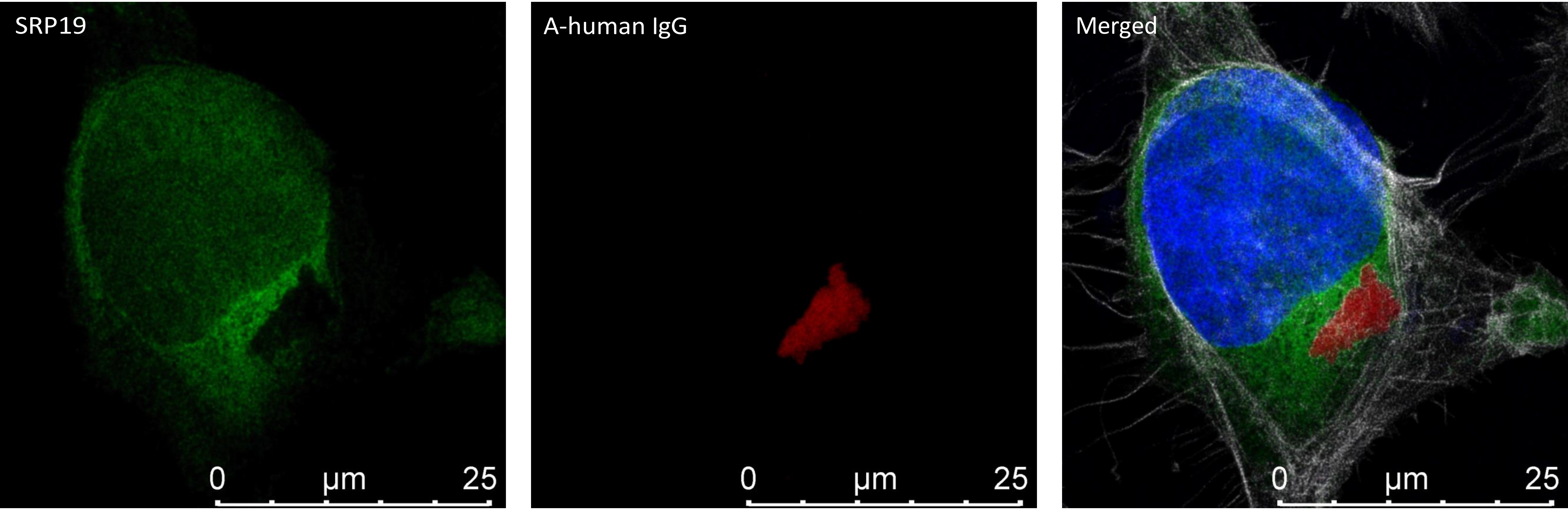

SJSA-1
DAPI, blue
Anti-human IgG, red
Anti-SRP19, green
Phalloidin, grey
Confocal microscope, 100X

M

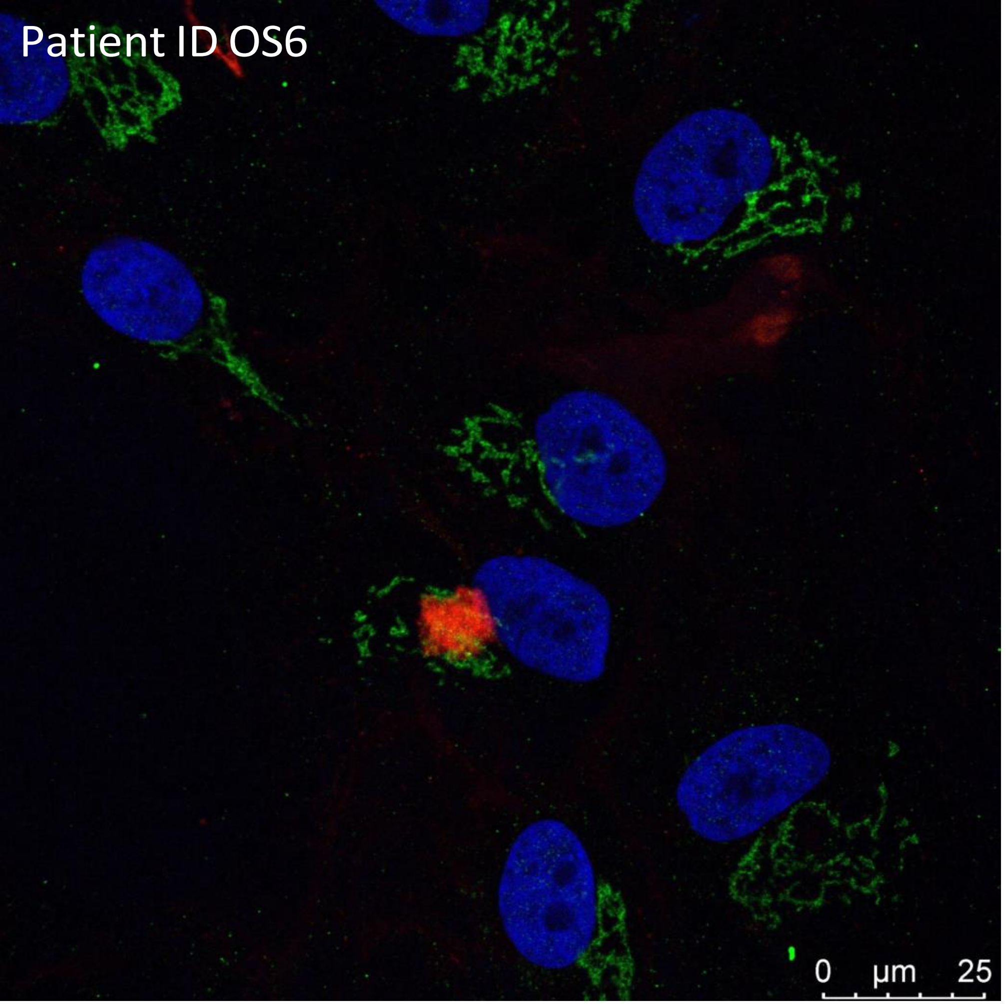

SJSA-1
DAPI, blue
Anti-human IgG, red
Anti-GOLGA1, green
Confocal microscope, 100X

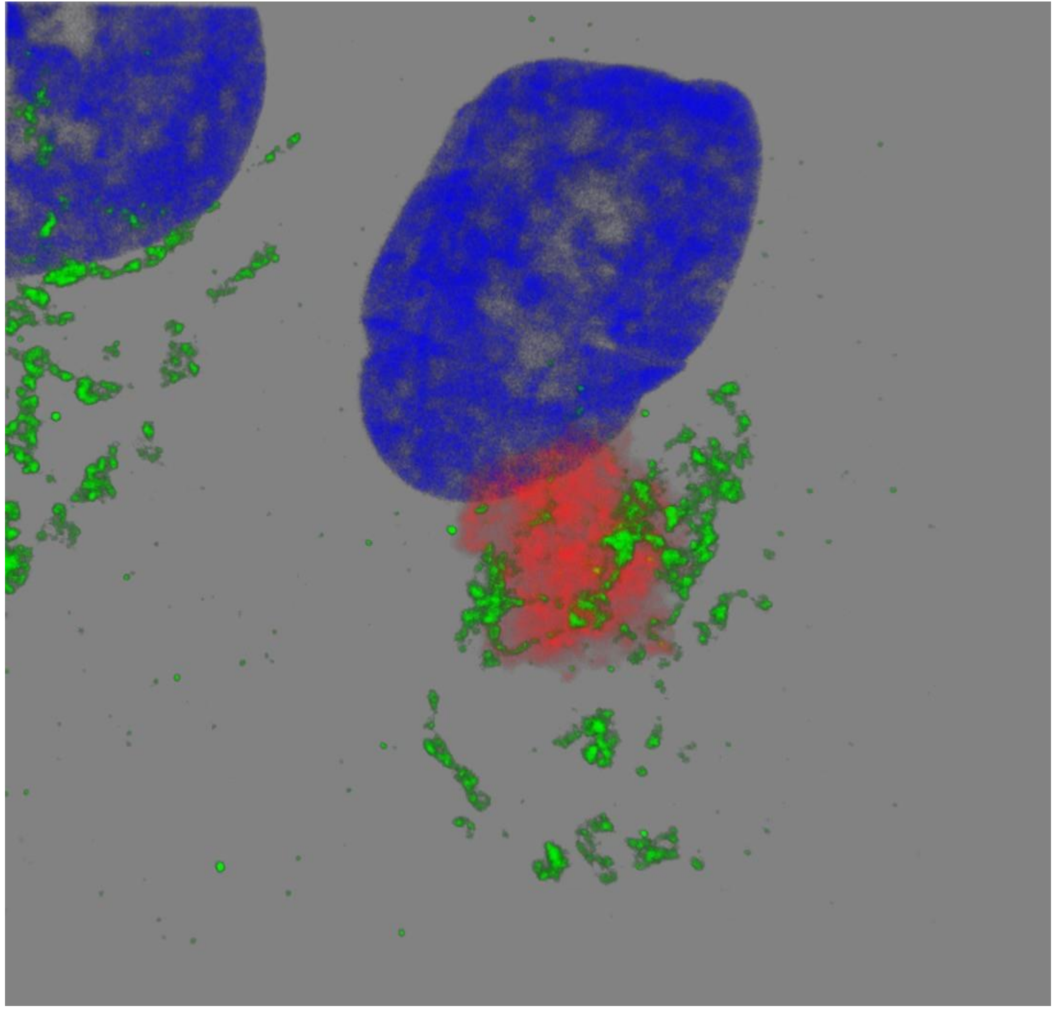

3D reconstraction of z-stack
mode acquired pictures
Confocal microscope, 100X

N

Supplementary figure S1

(O-Q) Representative pictures showing morphology of TISa pattern

Patient ID OS7

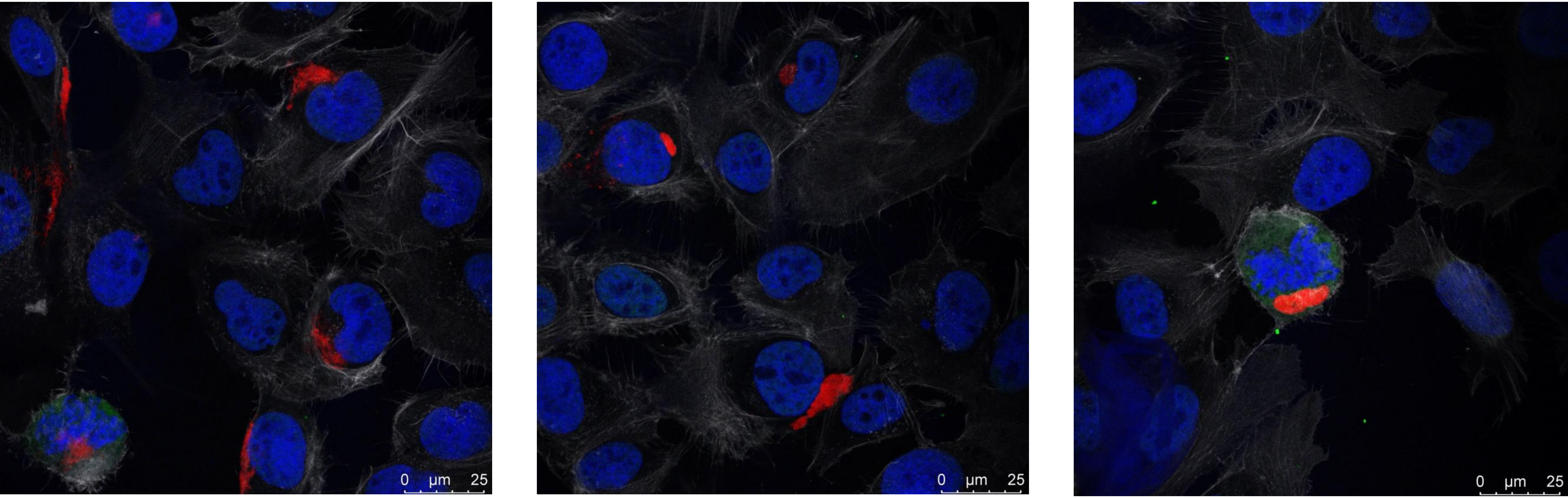

SJSA-1
DAPI, blue
Anti-human IgG, red
Phalloidin, grey
Confocal microscope, 100X

O

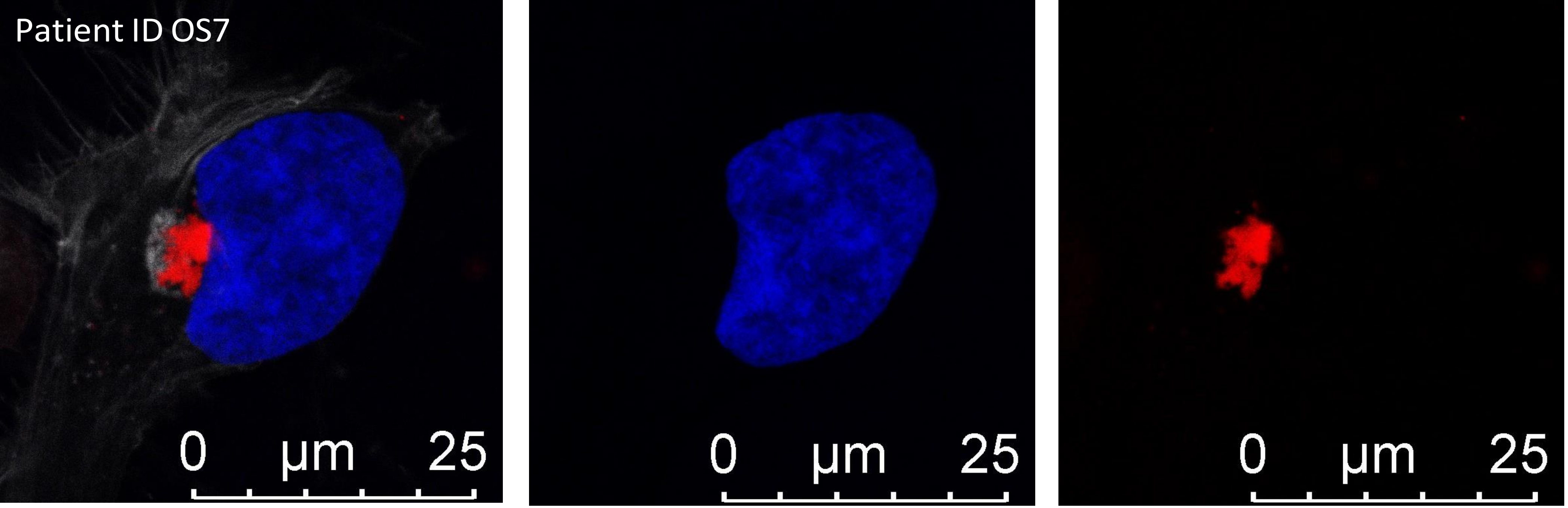

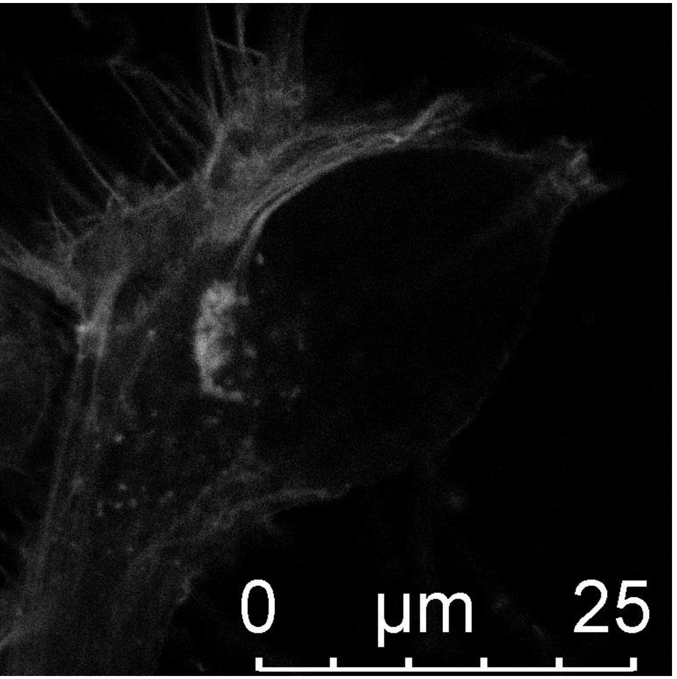

SJSA-1
DAPI, blue
Anti-human IgG, red
Phalloidin, grey
Confocal microscope, 100X

P

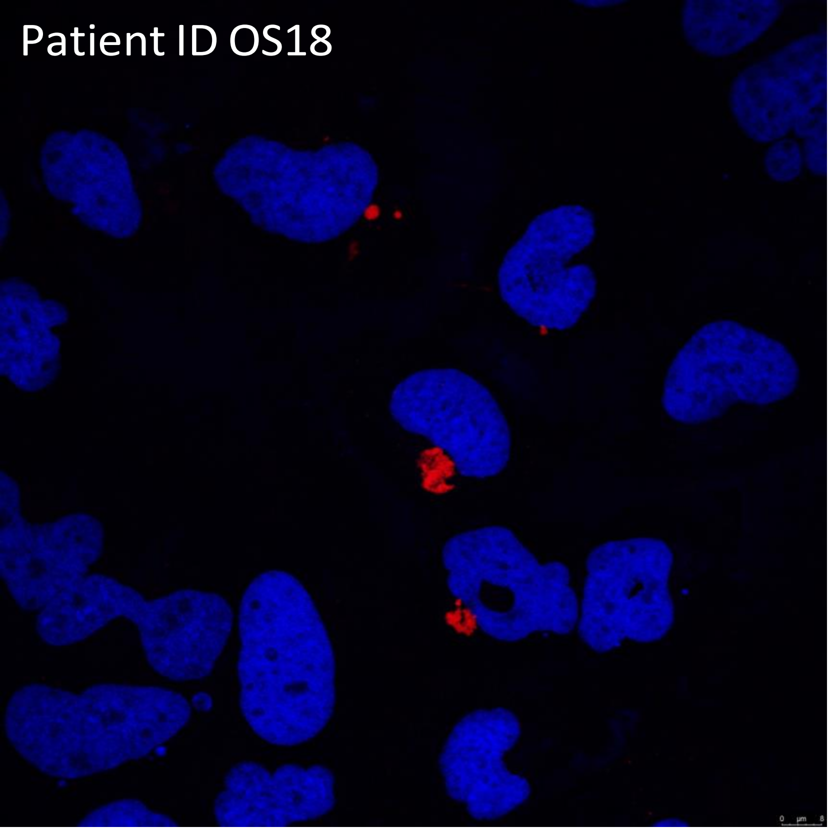

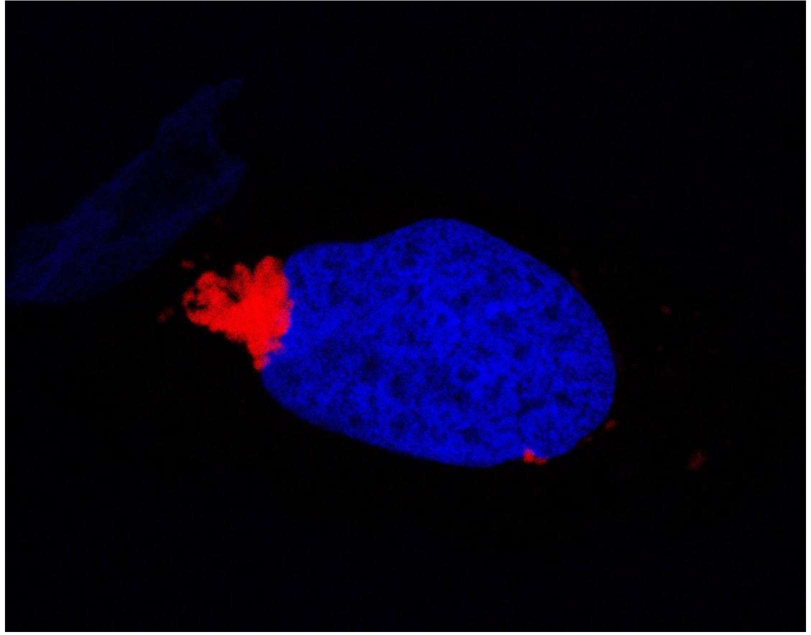

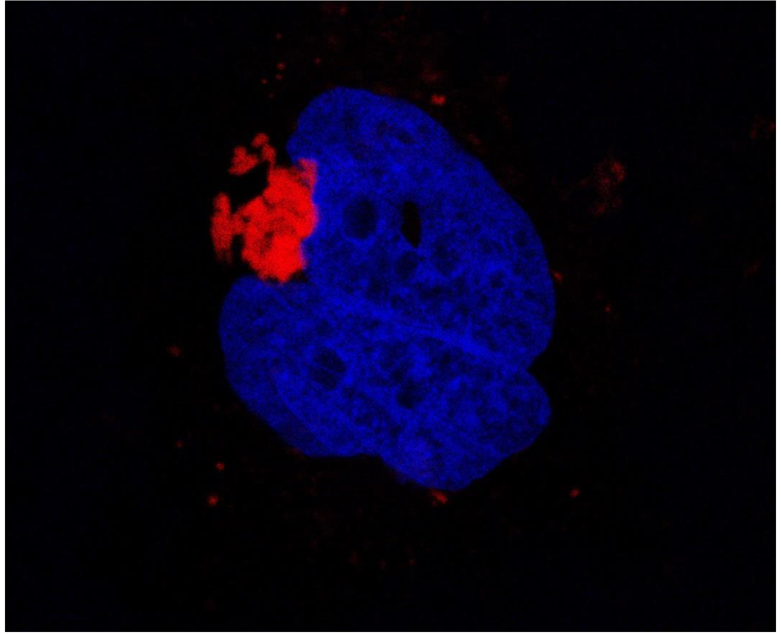

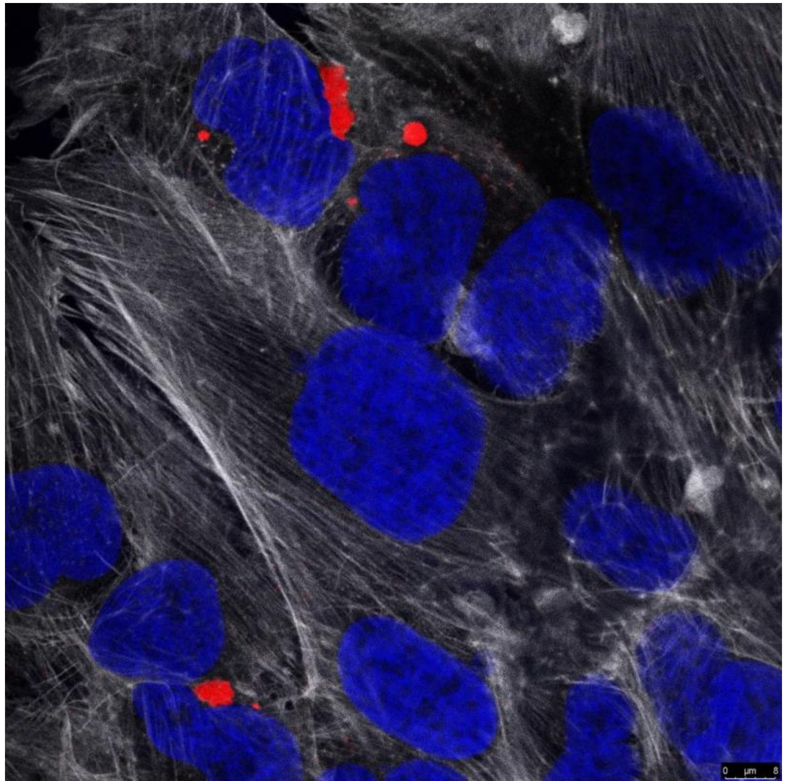

Cell line IOR15

DAPI, blue
Anti-human IgG, red
Phalloidin, grey

Q

Supplementary figure S1

(R-T) Representative pictures showing nuclear penetrating IgG

Patient ID OS22

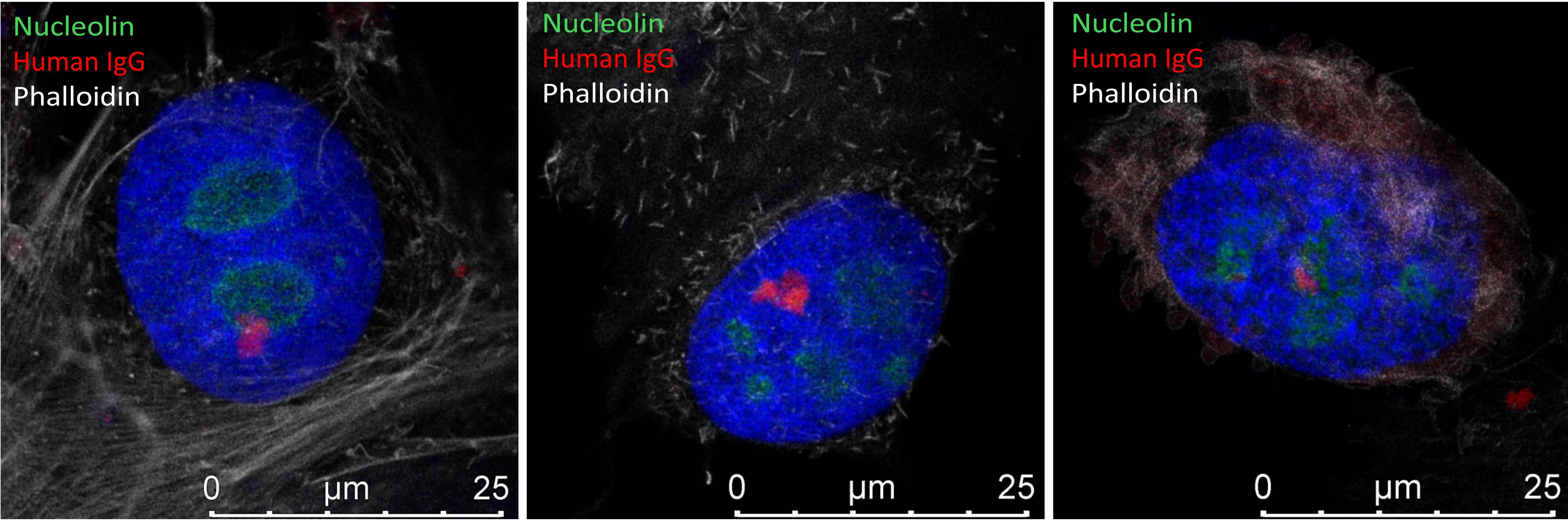

SJSA-1 cells blocked by cholchicine and incubated with patient serum

R

Patient ID AS1 (AngioSarcoma)

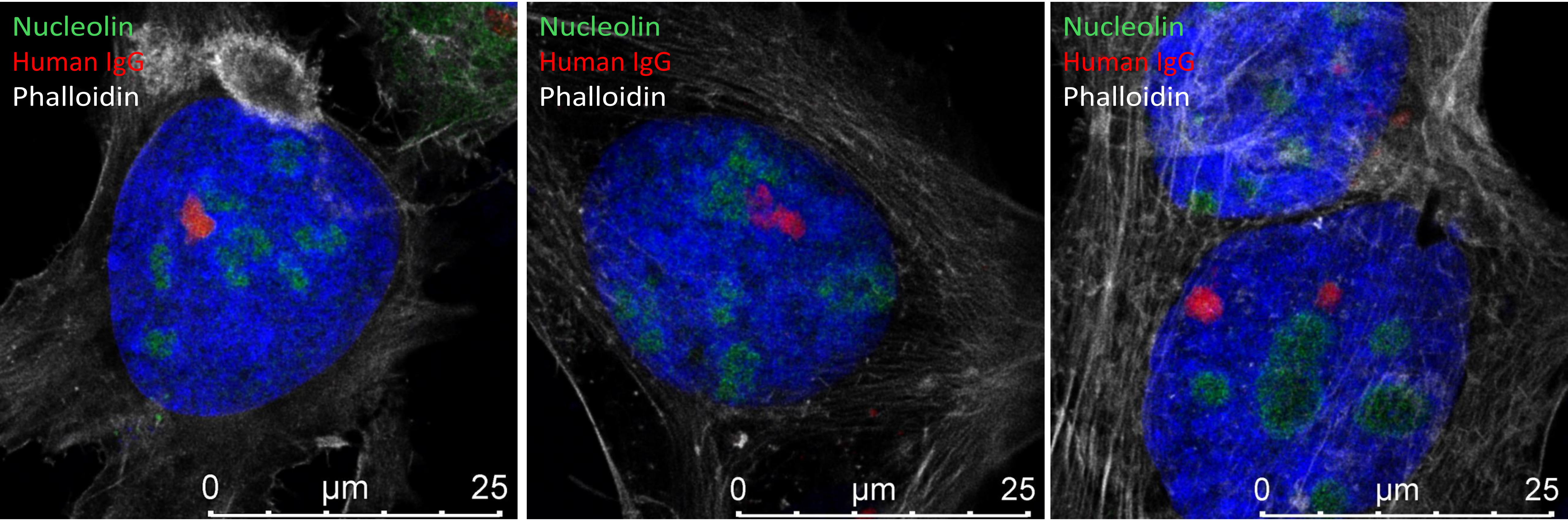

SJSA-1 cells blocked by cholchicine and incubated with patient serum

S

Patient ID OS8

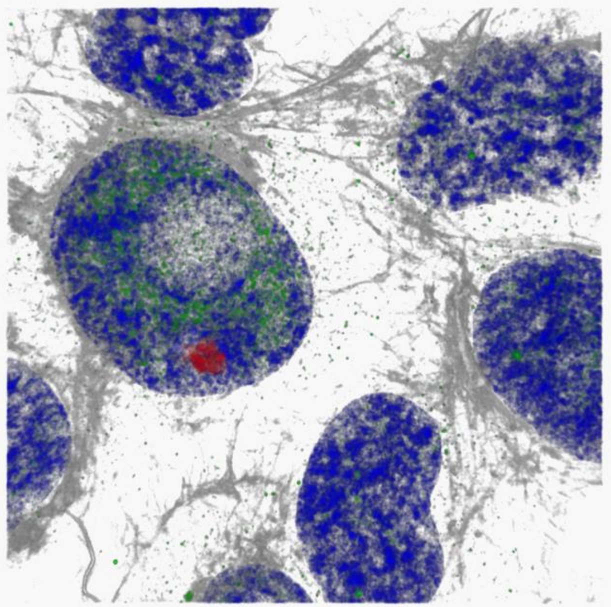

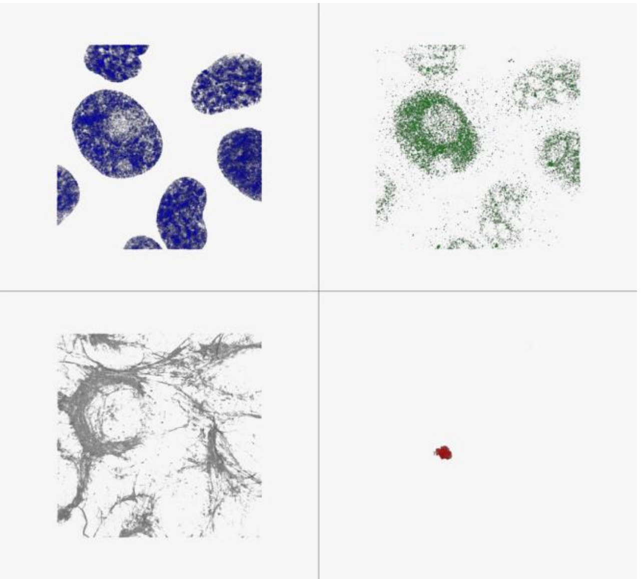

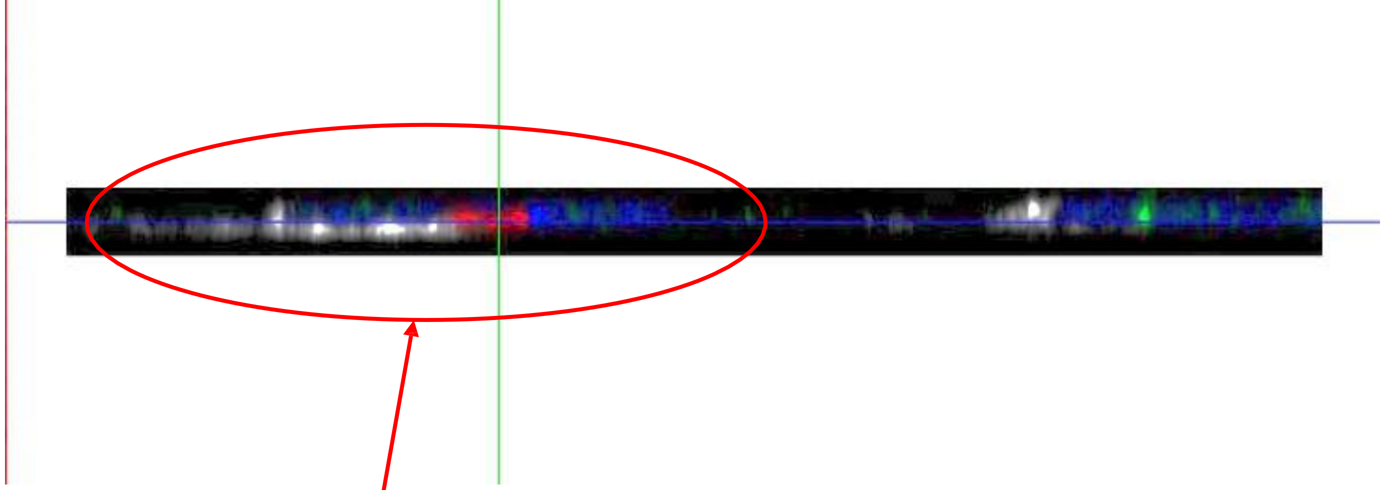

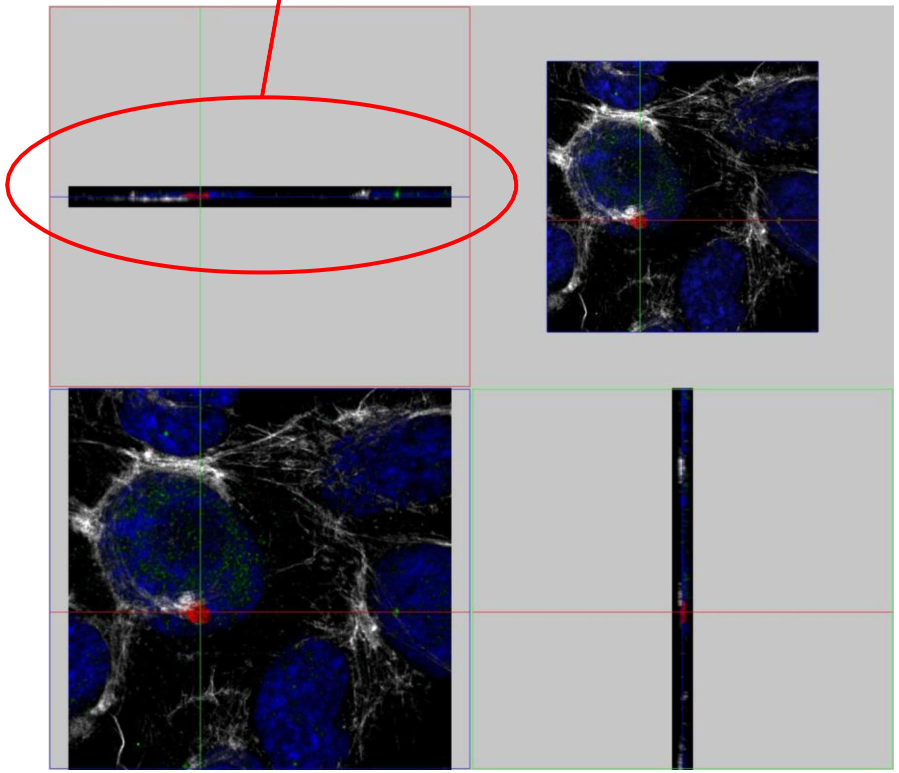

SJSA-1 cells

3D reconstruction of z-stack mode Confocal microscope 100X HNRNPC green

T

Supplementary figure S2

(A-K) Representative fluorescent images from different experiments investigating colocalization of penetrating IgG with candidate autoantigens

Patient ID AS1 (AngioSarcoma)

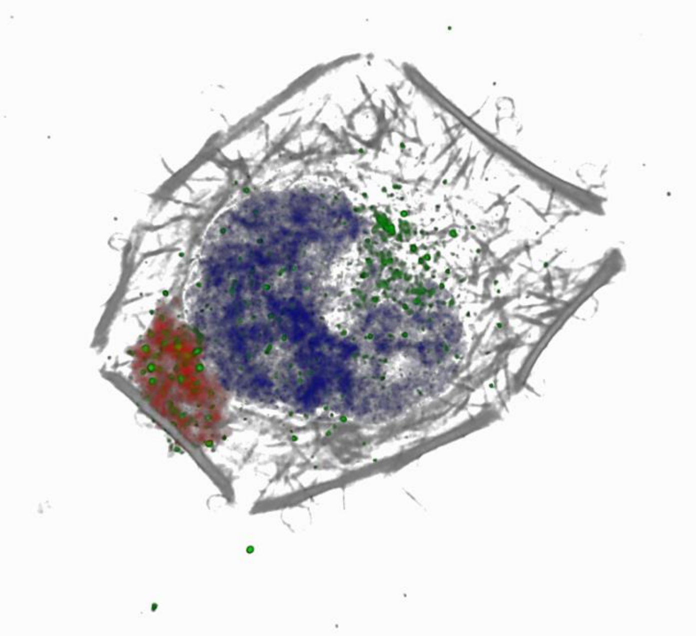

SJSA-1
DAPI, blue
Anti-human IgG, red
DHX58, green
Phalloidin, grey
Confocal, 100X

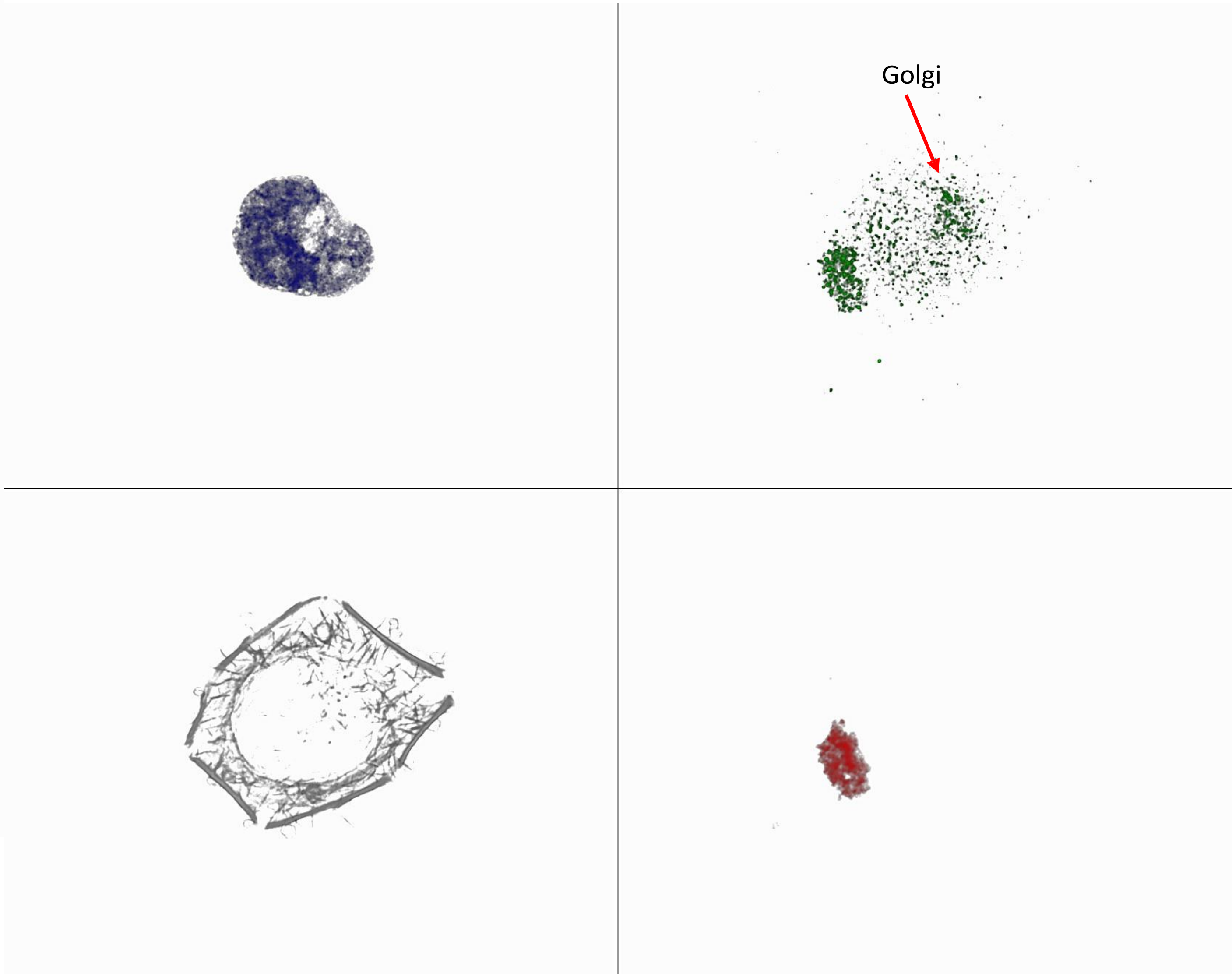

Note: DHX58 localizes to Golgi, to the nucleoplasm and cytosol, which can be clearly observed from this picture

3D structure of the specimen obtained through optical sectioning (z-stack).

A

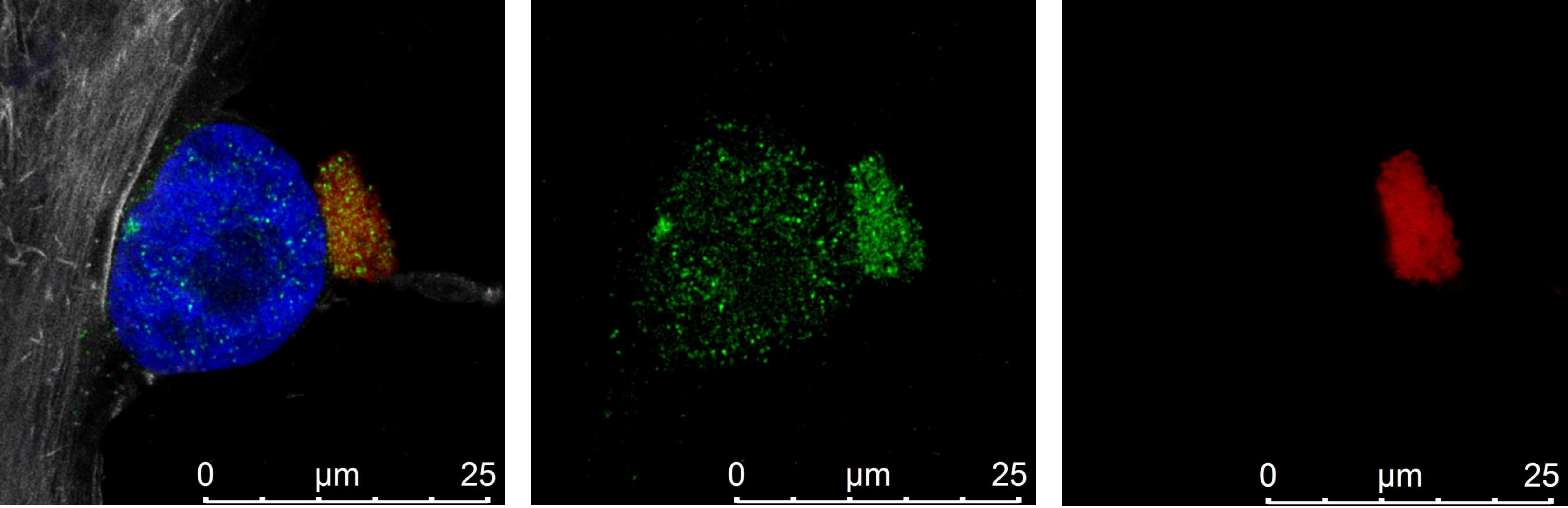

Patient AS1 (AngioSarcoma)
SJSA-1
DAPI, blue
Anti-human IgG, red
DHX58, green
Phalloidin, grey

B

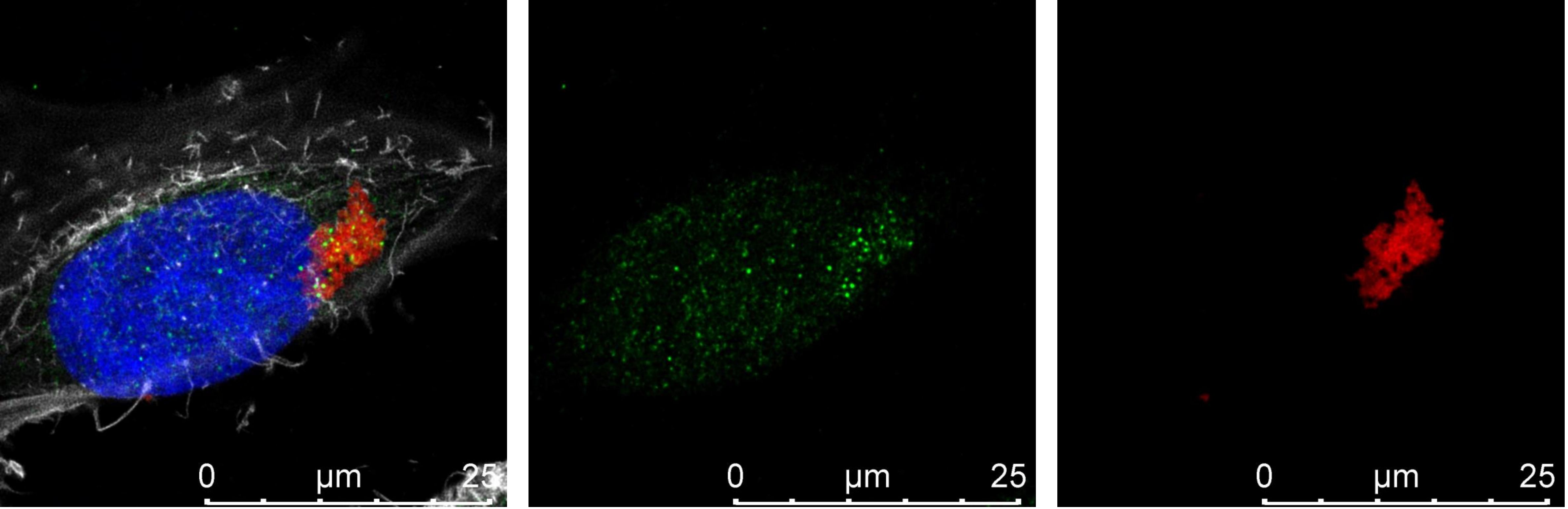

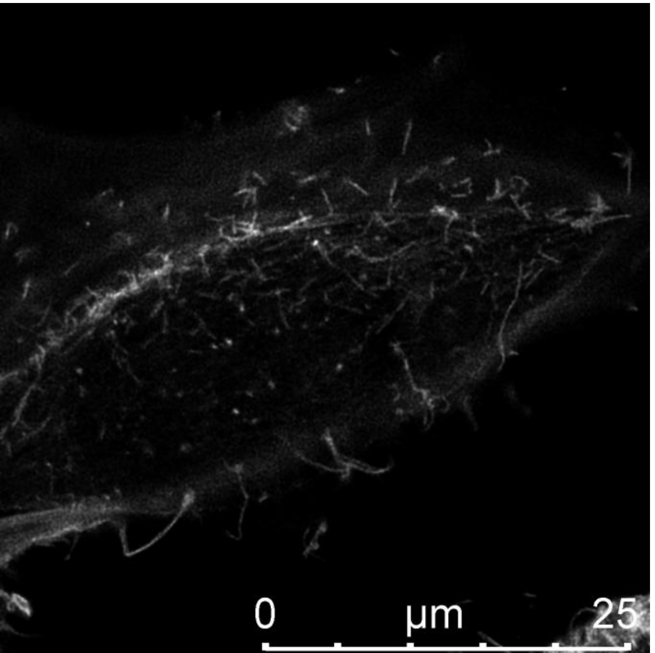

SJSA-1, **patient AS1** (AngioSarcoma)
DAPI, blue
Anti-human IgG, red
DHX58, green
Phalloidin, grey

C

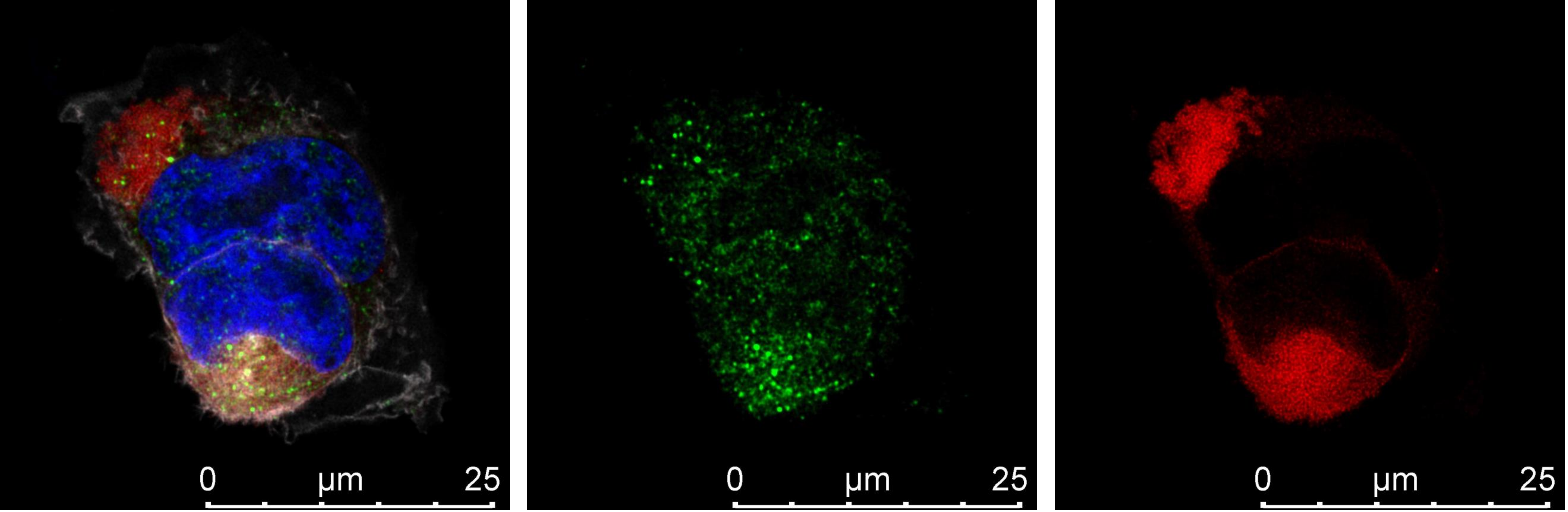

SJSA-1, **NBL12**
DAPI, blue
Anti-human IgG, red
RAD52, green

Phalloidin, grey

D

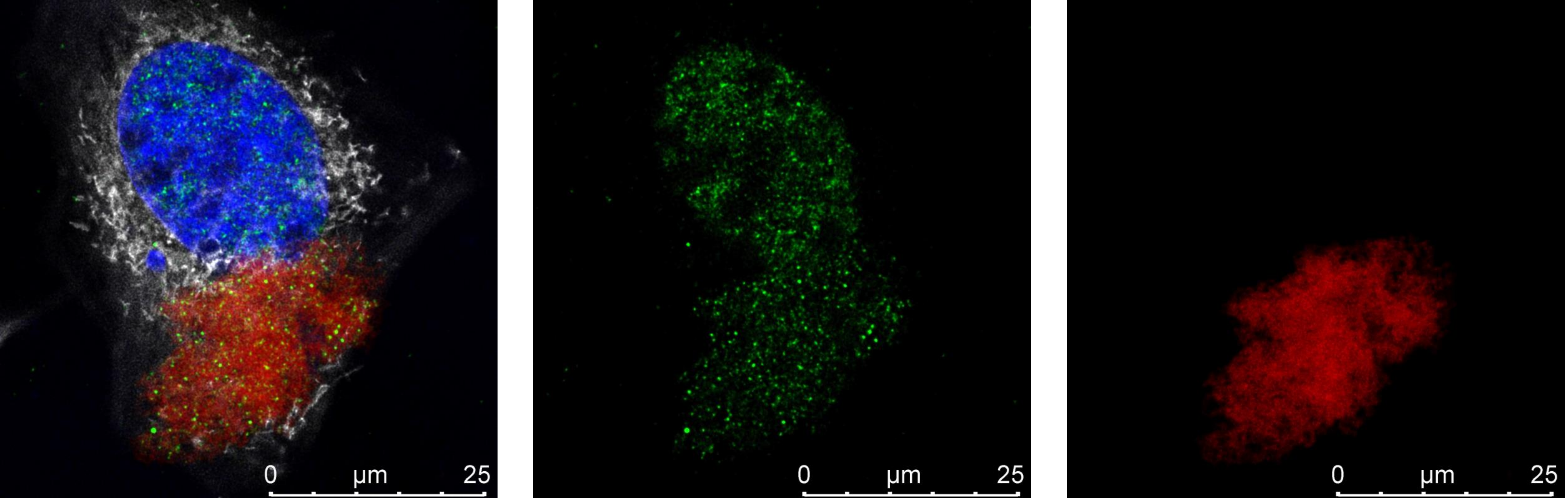

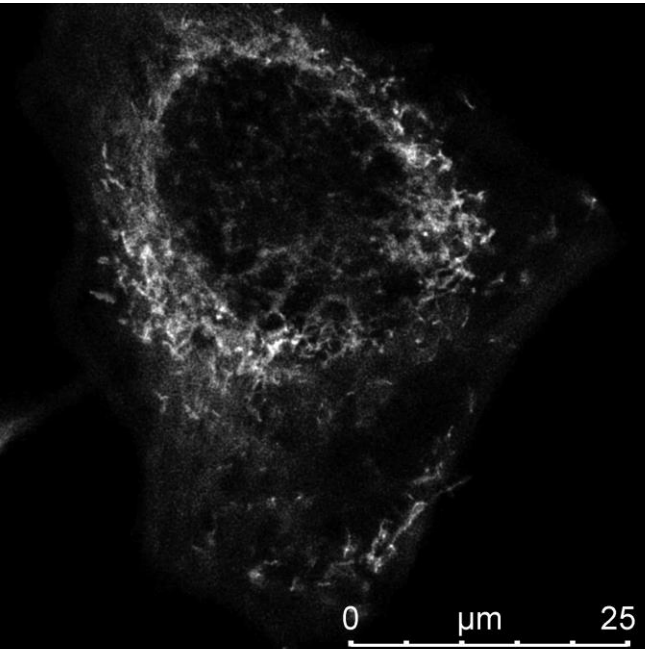

SJSA-1, **patient NBL2**
DAPI, blue
Anti-human IgG, red
RAD52, green

Phalloidin, grey

E

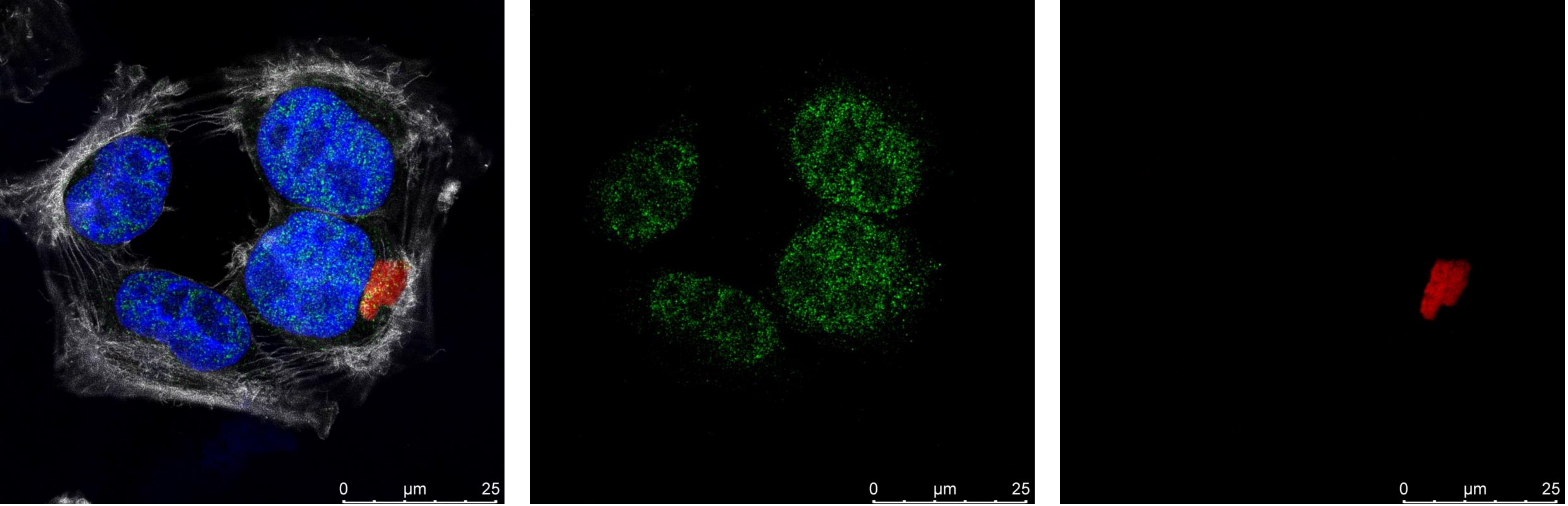

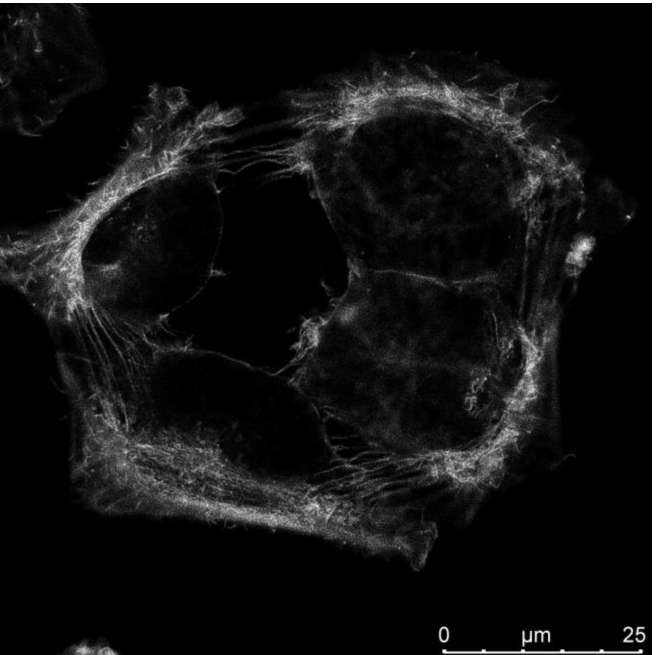

SJSA-1, **patient NBL2**
DAPI, blue
Anti-human IgG, red
RAD52, green

Phalloidin, grey

F

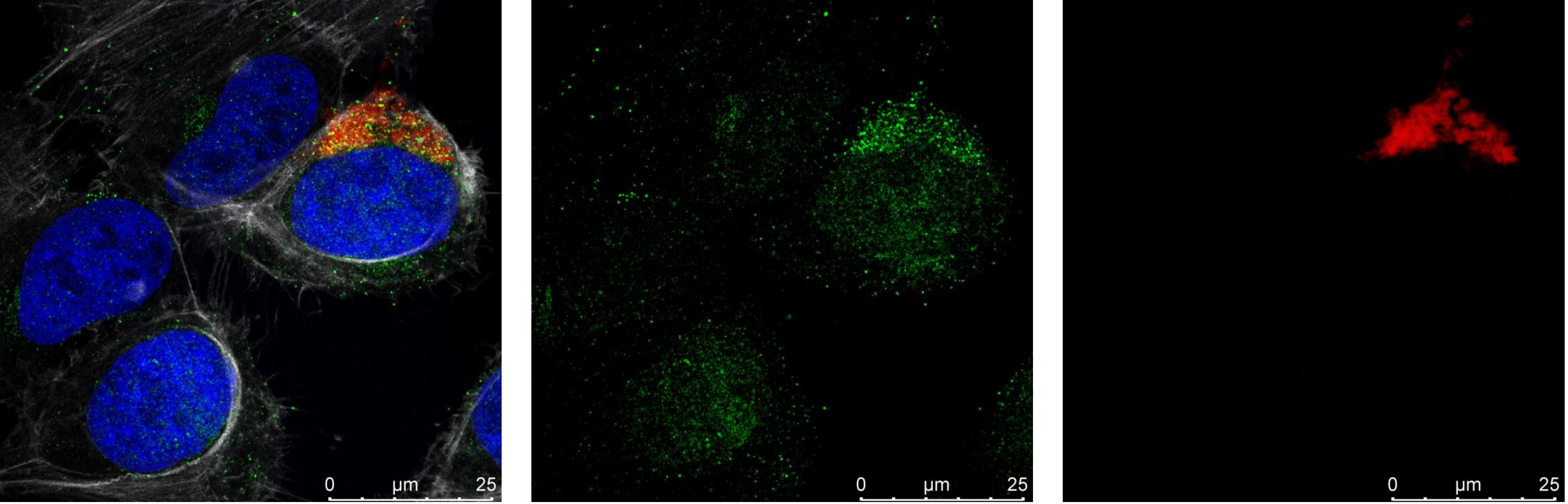

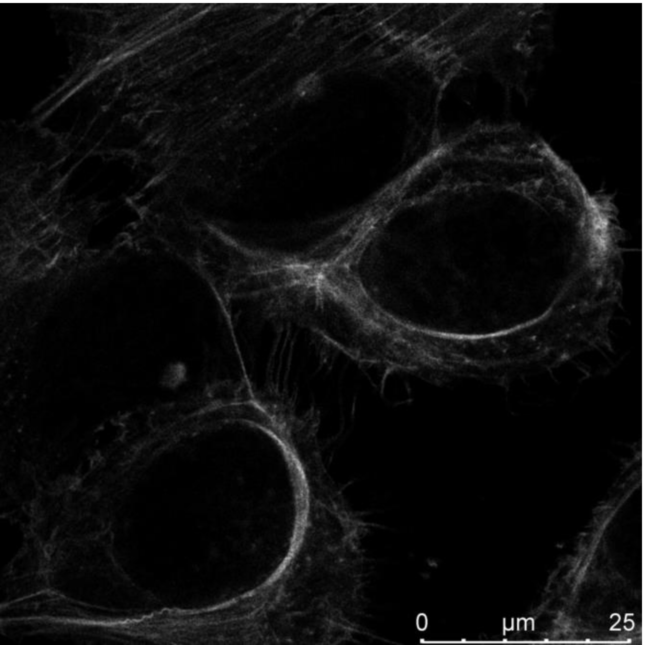

SJSA-1, **patient NBL2**
DAPI, blue
Anti-human IgG, red
CRAT, green

Phalloidin, grey

G

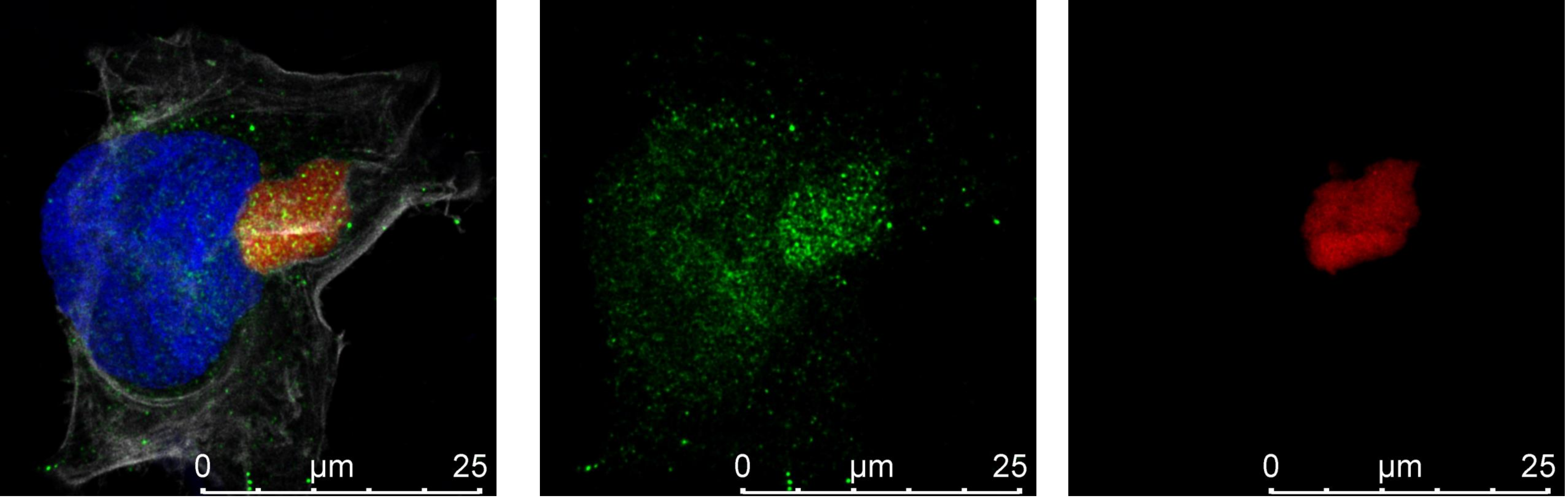

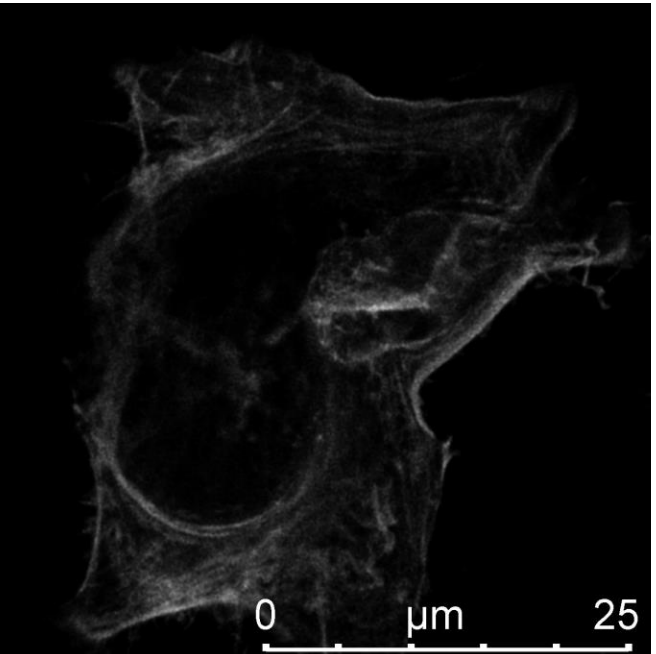

SJSA-1, **patient NBL2**
DAPI, blue
Anti-human IgG, red
CRAT, green

Phalloidin, grey

H

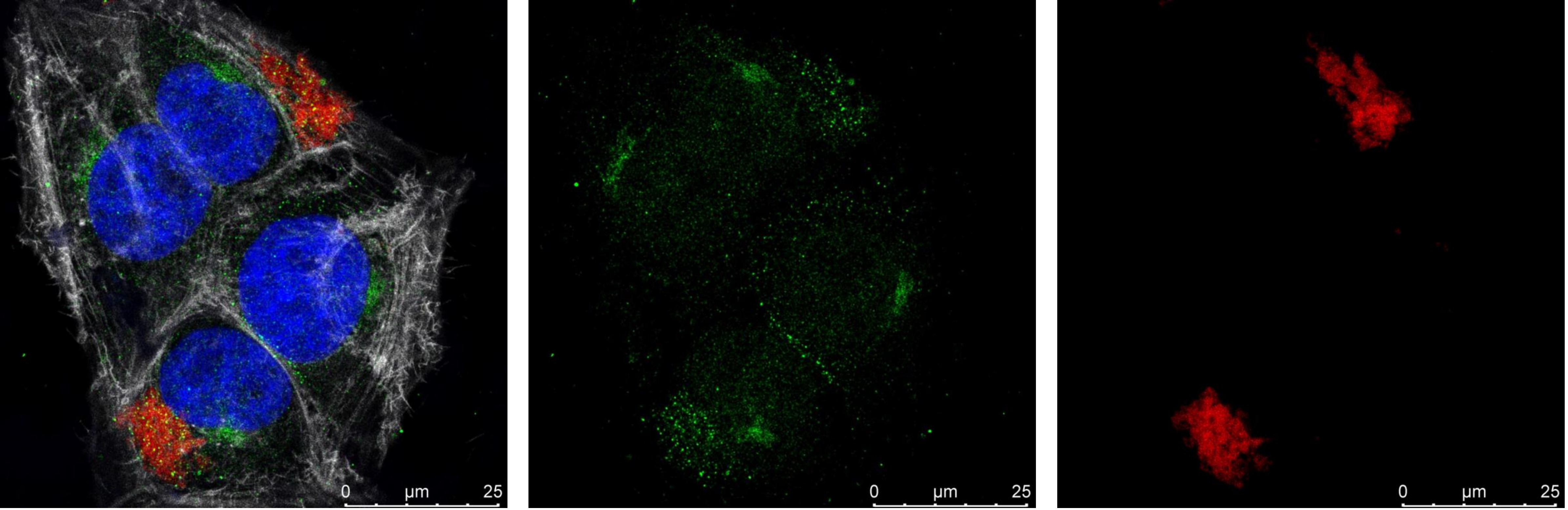

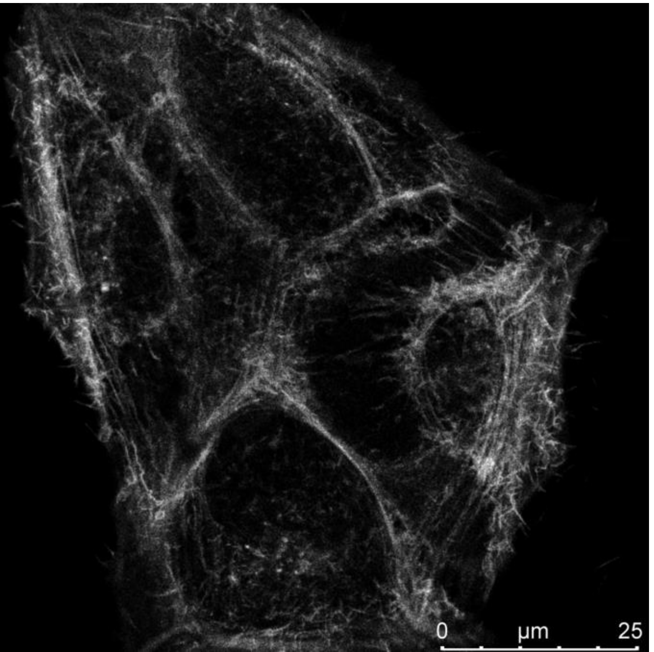

SJSA-1, **patient** N**BL2**
DAPI, blue
Anti-human IgG, red
CRAT, green
Phalloidin, grey
Confocal, 100X

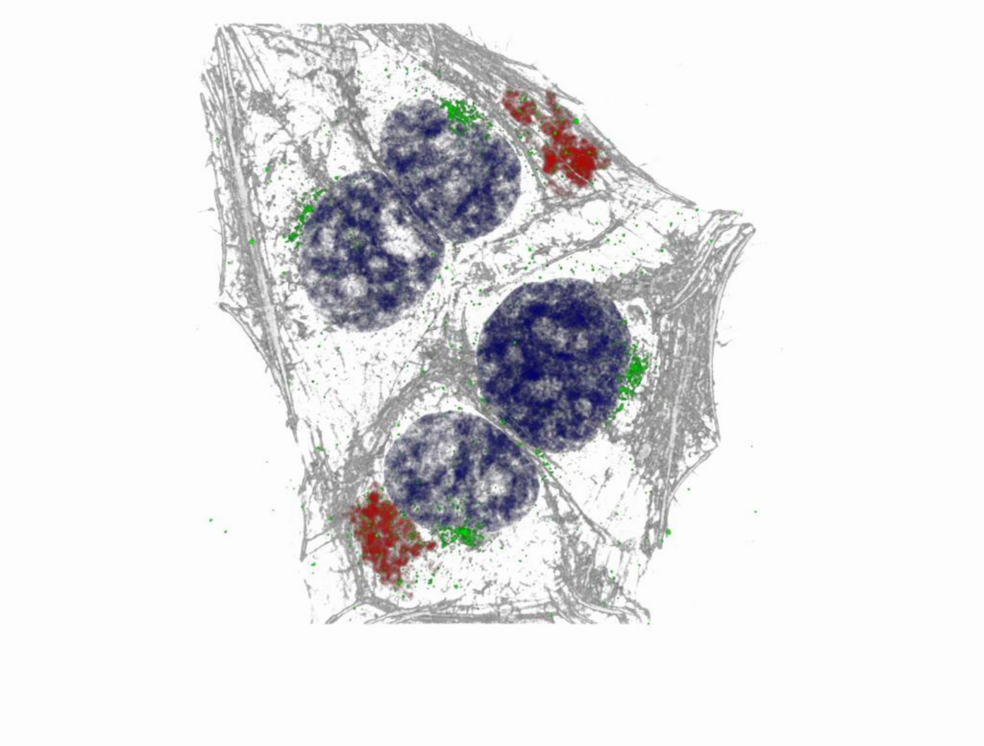

SJSA-1, **patient NBL2**
DAPI, blue
Anti-human IgG, red
CRAT, green
Phalloidin, grey
Confocal, 100X

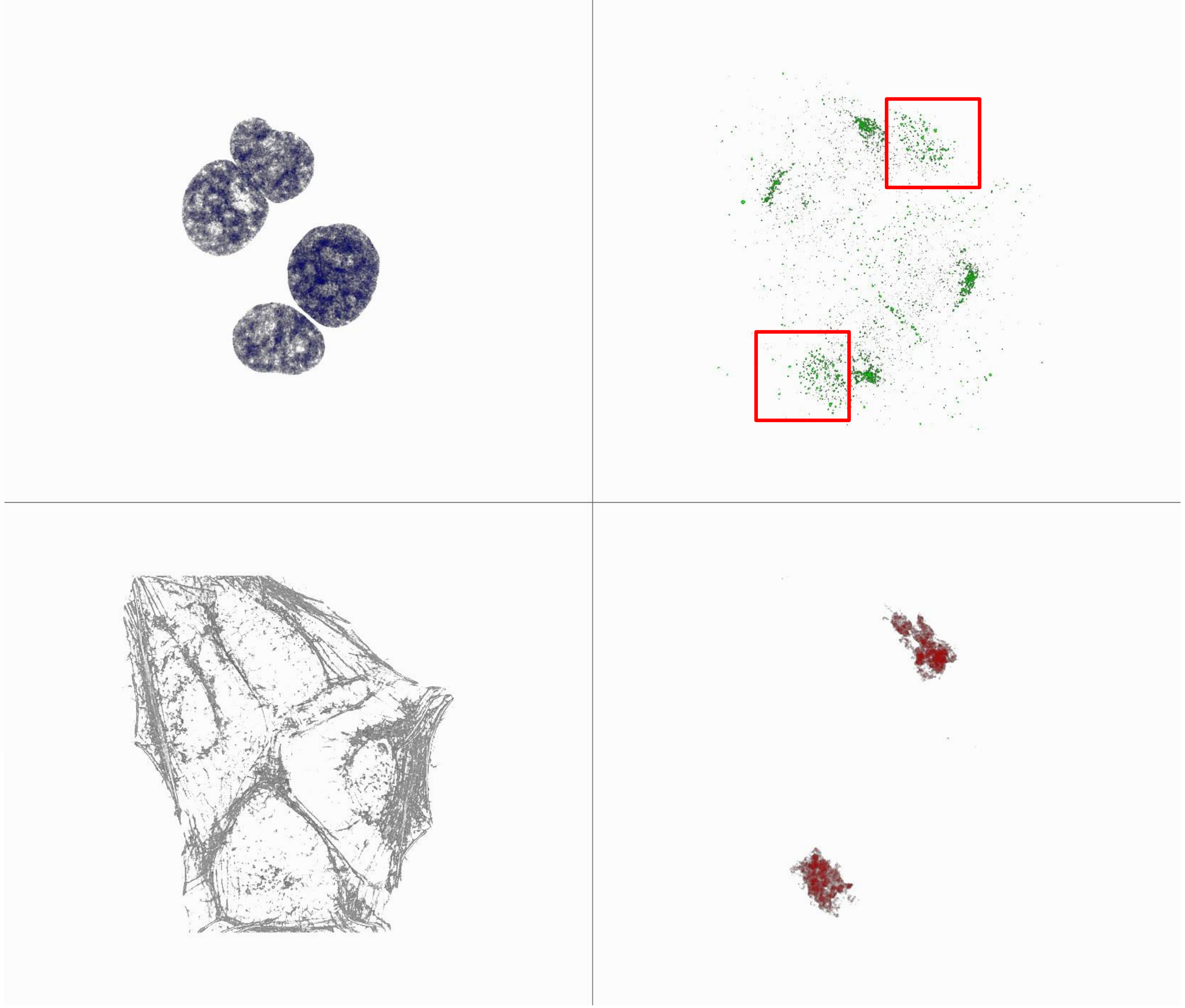

3D structure of the specimen obtained through optical sectioning (z-stack). Red squares indicate CRAT trapped inside TISa.

J

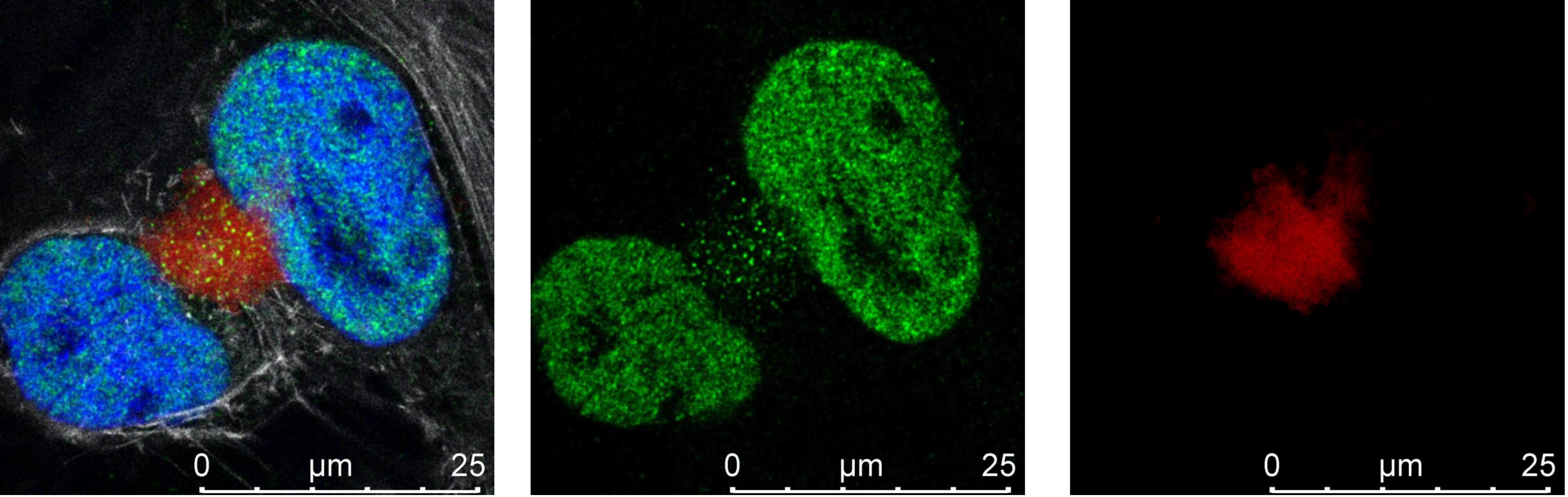

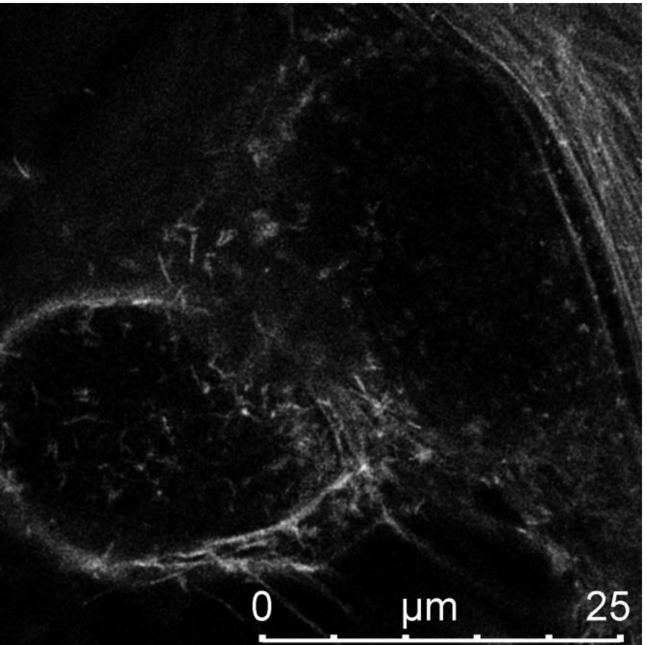

SJSA-1, **patient Y1C2**
DAPI, blue
Anti-human IgG, red
NHRNPC, green
Phalloidin, grey

K

Supplementary figure S2

(L-M) Representative fluorescent images of dividing cells stained for human IgG and candidate autoantigens

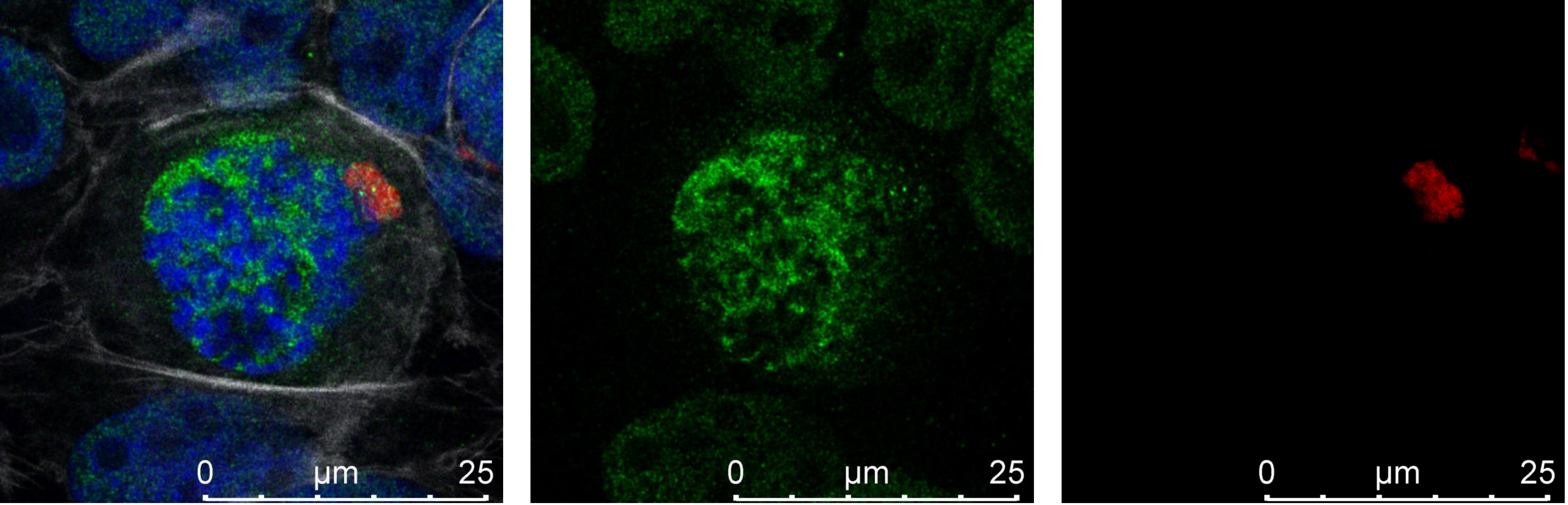

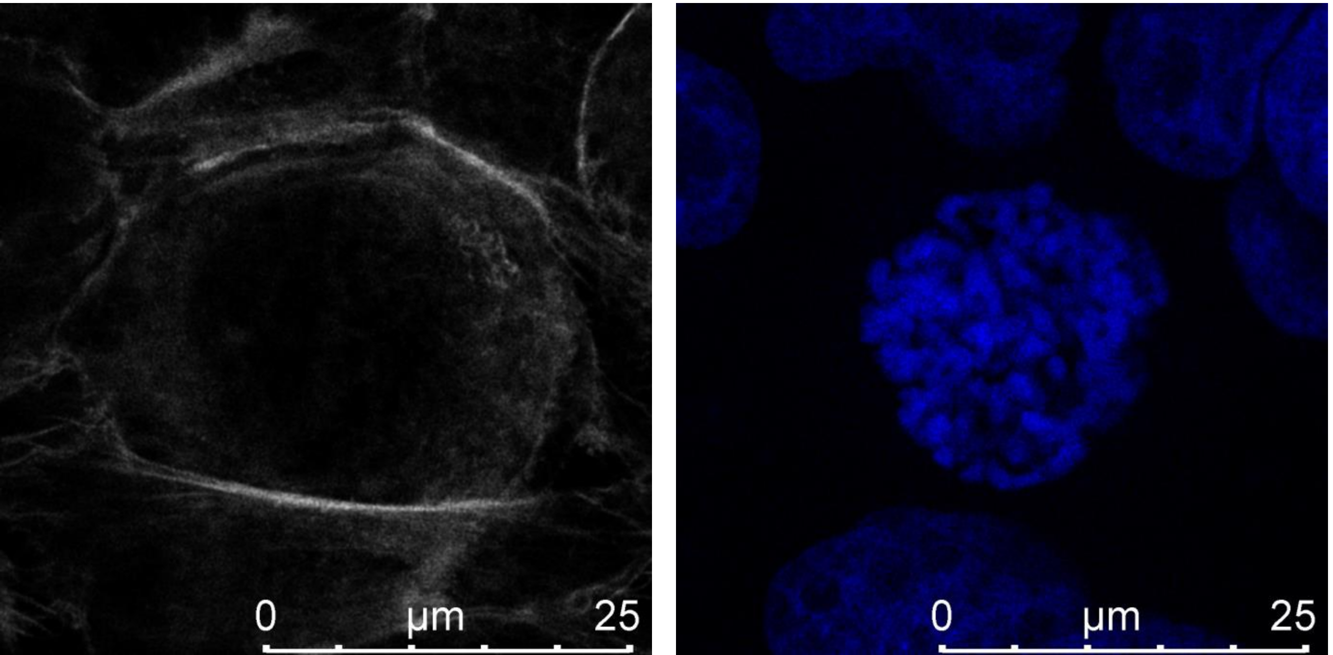

SJSA-1, **patient Y1C2**
DAPI, blue
Anti-human IgG, red
NHRNPC, green
Phalloidin, grey

L

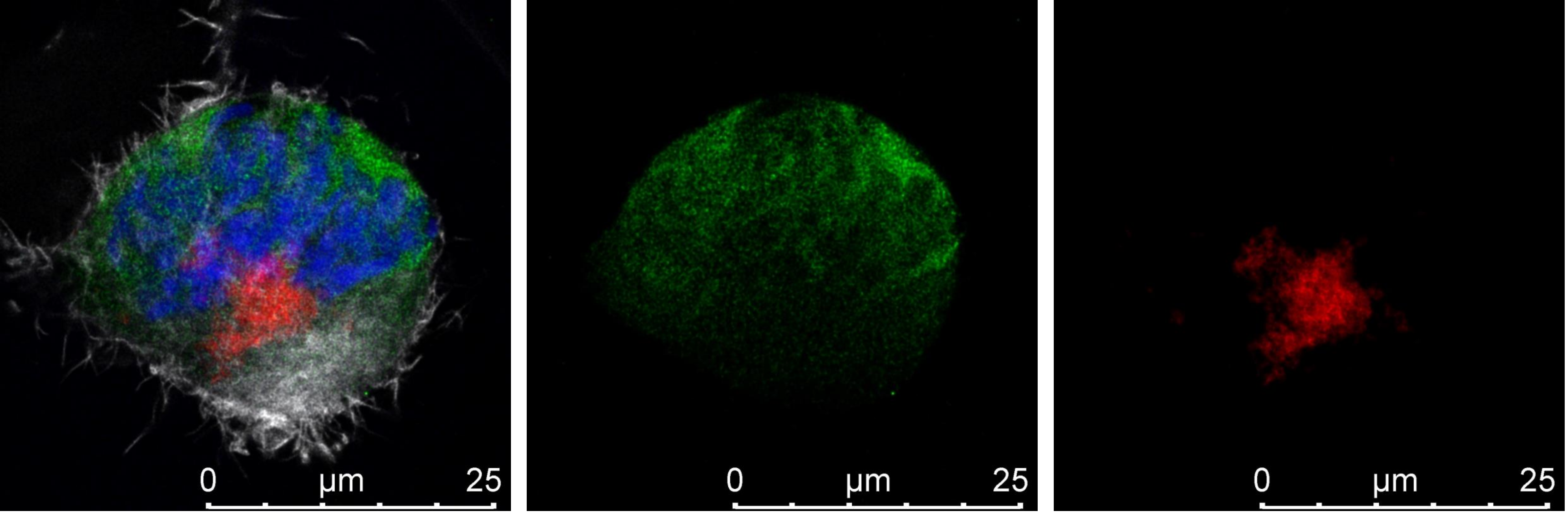

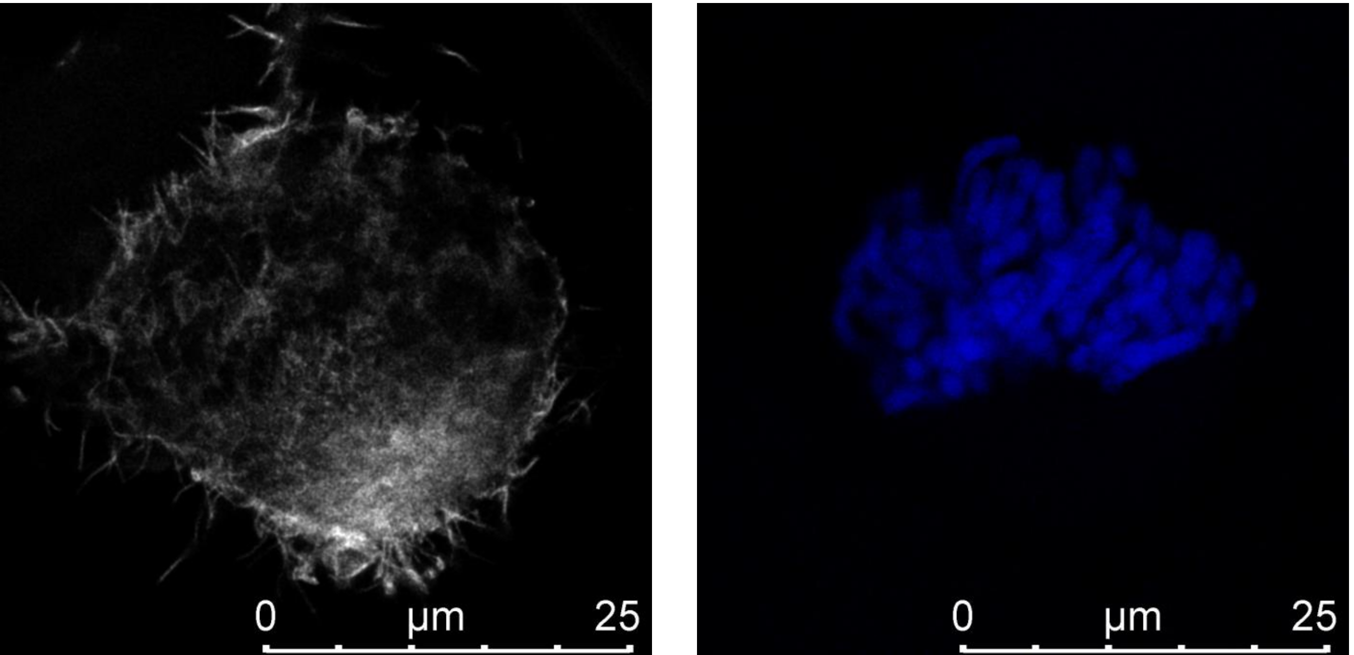

SJSA-1, **patient Y1C2**
DAPI, blue
Anti-human IgG, red
NHRNPC, green
Phalloidin, grey

M
