# Supplementary material for: Pathogenic autoantibodies targeting DNA repair proteins are shared by cancer patients and healthy children later diagnosed with cancer
Source: Front Immunol. 2026 Oct 14;17:1911932. doi: 10.3389/fimmu.2026.1911932 (PMC13713722; doi:10.3389/fimmu.2026.1911932)
Supplement: Supplementary file 1 [file DataSheet1.zip › Supplementary Data/Supplementary Figure S3.pdf]

A

Cell cycle phases distribution

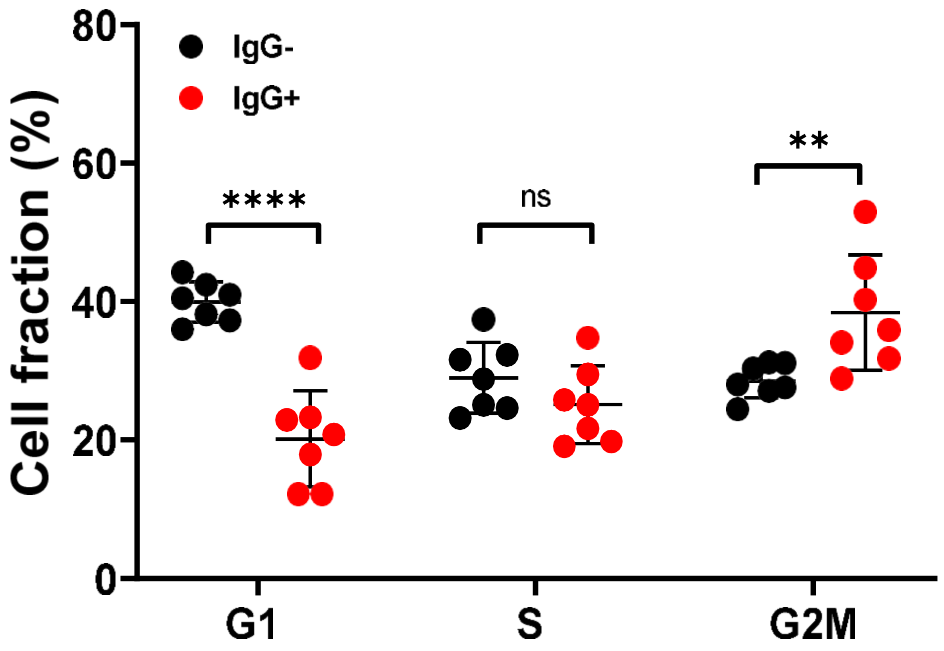

The representative dot graph shows the fraction of IgG-positive cells (incubated with control serum) in different cell cycle phases. Cell cycle analysis was done using Dean-Jett Fox algorithms in FlowJo (10.8.1). Statistical analysis was done by Two-way ANOVA (Sidak's multiple comparison test) showing the mean±SD of seven replicates. ****$P \leq 0.0001$; **$P \leq 0.01$;ns non-significant

B

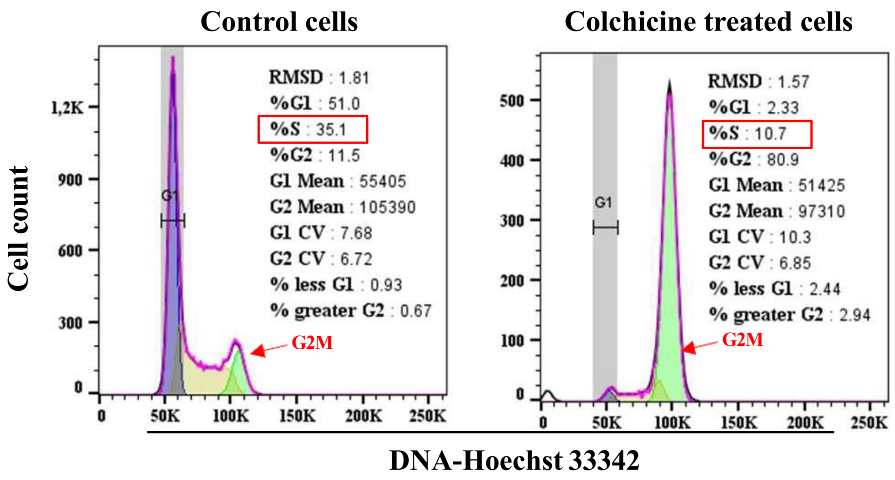

The representative histogram shows the cells cycle block and gating. Cells treated with colchicine (25 ng/ml) were blocked in G2M.

C

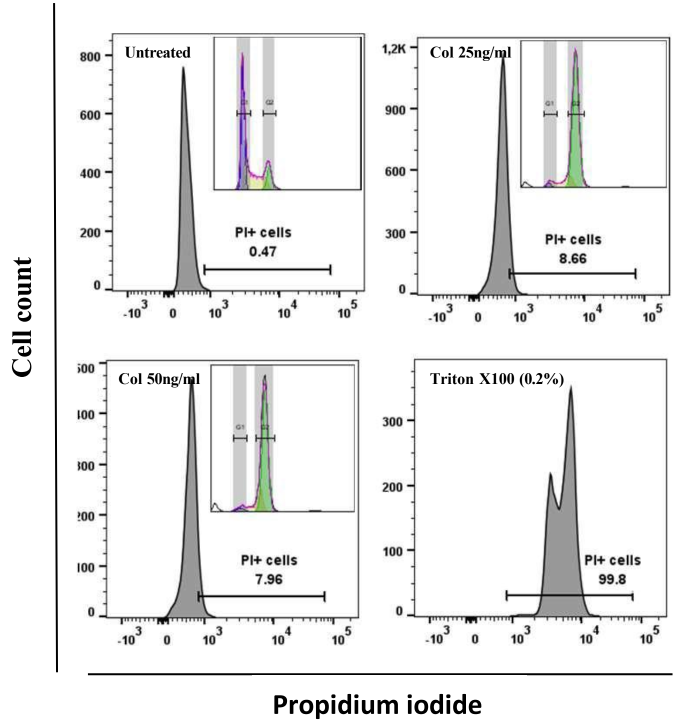

Representative histogram shows an increase in propidium iodide positive cells (7-8%) after Colchicine (Col) treatment. Propidium iodide was used for live/dead staining.

Triton X100 was used as a positive control
