# Supplementary material for: Pathogenic autoantibodies targeting DNA repair proteins are shared by cancer patients and healthy children later diagnosed with cancer
Source: Front Immunol. 2026 Oct 14;17:1911932. doi: 10.3389/fimmu.2026.1911932 (PMC13713722; doi:10.3389/fimmu.2026.1911932)
Supplement: Supplementary file 1 [file DataSheet1.zip › Supplementary Data/Table S1.pdf]

**Cohorts**	**Age span (years)**	**Number**
**Controls**	**0-69**	**141**
Blood donors and volunteers	22-69	81
School children	7-14	39
Pediatric trauma patients	0,6-11	21
**Patient cohorts**	**0,3-89**	**175**
Neuroblastoma	0,3-2	10
Glioma	0,2-16	11
Wilm's tumour	0,3-8	10
Leukemia (ALL)	1-85	24
Osteosarcoma	2-76	59
Rhabdomyosarcoma	0,1-82	28
Liposarcoma	39-89	16
Undifferentiated pleomorphic sarcoma	38-89	12
Embryonic cancers:	**0,1-0,8**	**5**
Atypical teratoid rhabdoid tumour	0,1-0,8	3
Hepatoblastoma	0,3	1
Pulmonary blastoma	0,5	1

Table S1. Patient and control cohorts analyzed by proteome arrays. 10 patients with osteosarcoma, 5 with rhabdomyosarcoma, and 2 with liposarcoma were treated with chemotherapy before blood sampling.
