# Supplementary material for: Pathogenic autoantibodies targeting DNA repair proteins are shared by cancer patients and healthy children later diagnosed with cancer
Source: Front Immunol. 2026 Oct 14;17:1911932. doi: 10.3389/fimmu.2026.1911932 (PMC13713722; doi:10.3389/fimmu.2026.1911932)
Supplement: Supplementary file 1 [file DataSheet1.zip › Supplementary Data/Table S3.pdf]

Cancer	NBL	Wilm's	Glioma	ALL	RMS	OS
#cases	946	343	688	2647	208	282
Protein						
ACOT12	0.11		0.15	0.08		
ADRM1					0.96	
ALKBH7						
APBB1				0.11		0.35
CPNE6	0.53		0.29	0.04		
CRAT			0.29	0.08		0.35
DHX58	0.32					0.71
DMC1					0.48	0.71
DMD			0.73	0.98	1.44	1.77
HMGN1			0.29	0.04		
HSPA13			0.15	0.08		
MSH5	0.11		0.15			
NUMA1	0.95	0.29	1.16	0.34	0.48	0.71
PLCD4	0.11		0.15	0.08		
POLB				0.23		
POLR2H					0.48	
RAD52						0.35
RCHY1						
SRP19	0.11					0.35
TCEB2						
TP53I3				0.04		
UBE2N			0.15			

Fraction (%) of samples of each cancer type in the Pecan database with mutations affecting protein structure (missense, splicing, nonsense, indels, frame shifts,fusions) Ref

https://pecan.stjude.cloud

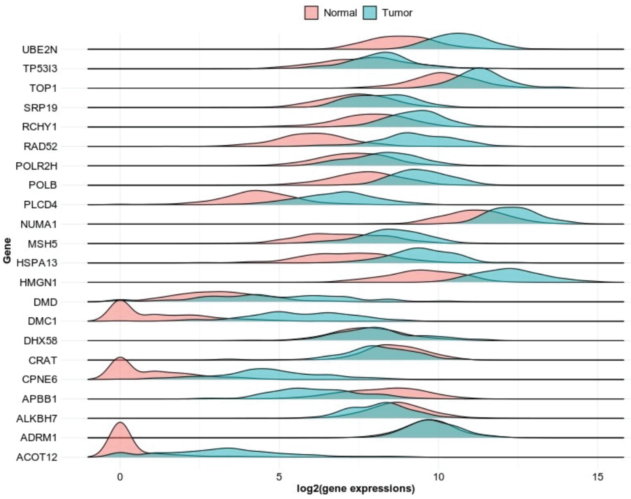

Acute Lymphatic leukemia

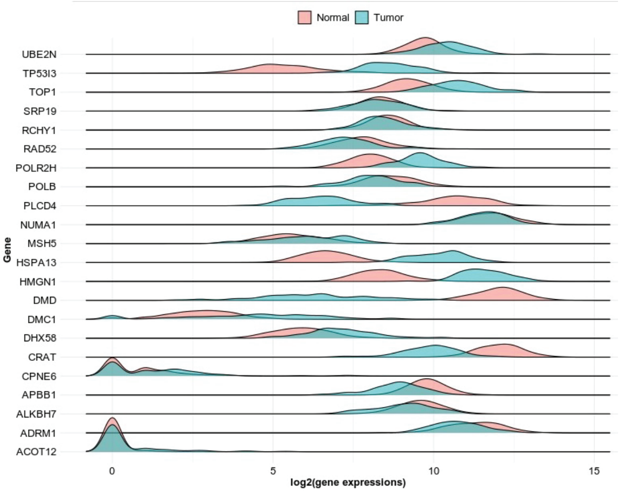

Osteosarcoma

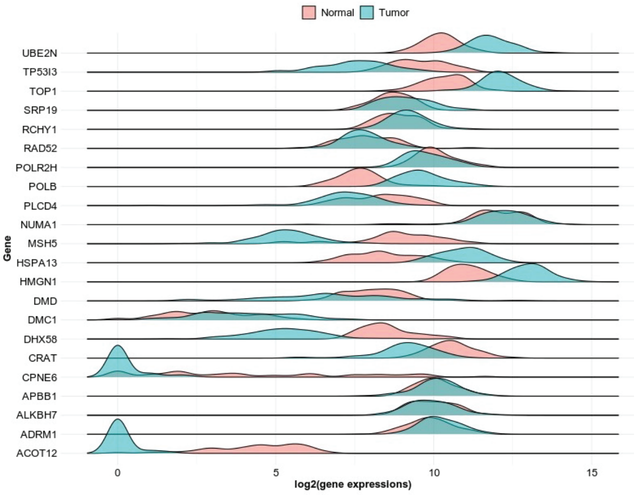

Rhabdoid tumour

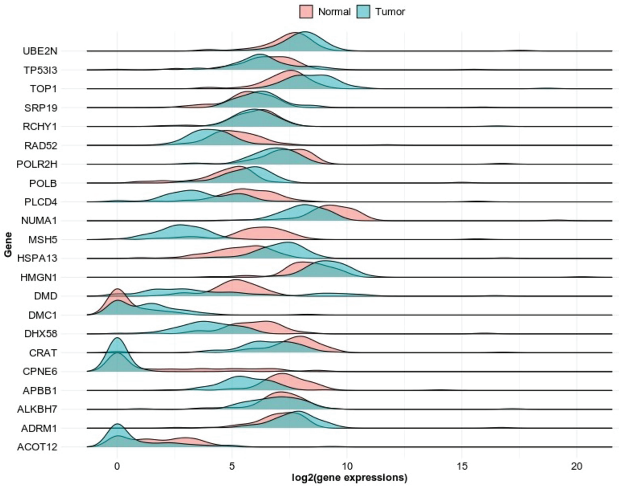

Wilm’s Tumour

Tumour/Normal plots from https://tnmplot.com/analysis/ showing expression of our core DNA repair antigens
